# Krüppel-like factor 2 controls IgA plasma cell compartmentalization and IgA responses

**DOI:** 10.1038/s41385-022-00503-0

**Published:** 2022-03-28

**Authors:** Jens Wittner, Sebastian R. Schulz, Tobit D. Steinmetz, Johannes Berges, Manuela Hauke, William M. Channell, Adam F. Cunningham, Anja E. Hauser, Andreas Hutloff, Dirk Mielenz, Hans-Martin Jäck, Wolfgang Schuh

**Affiliations:** 1 Division of Molecular Immunology, Department of Internal Medicine 3, Nikolaus-Fiebiger Center, University Hospital Erlangen, Friedrich-Alexander-University Erlangen-Nürnberg, Erlangen, Germany; 2 Institute of Immunology and Immunotherapy, University of Birmingham, Birmingham, UK; 3 Department of Rheumatology and Clinical Immunology, Charité - Universitätsmedizin Berlin, corporate member of Freie Universität Berlin and Humboldt-Universität zu Berlin, Berlin, Germany; 4 Deutsches Rheuma-Forschungszentrum (DRFZ), a Leibniz Institute, Berlin, Germany; 5 Institute of Immunology and Institute of Clinical Molecular Biology, University Hospital Schleswig-Holstein, Kiel, Germany

## Abstract

Krüppel-like factor 2 (KLF2) is a potent regulator of lymphocyte differentiation, activation and migration. However, its functional role in adaptive and humoral immunity remains elusive. Therefore, by using mice with a B cell-specific deletion of KLF2, we investigated plasma cell differentiation and antibody responses. We revealed that the deletion of KLF2 resulted in perturbed IgA plasma cell compartmentalization, characterized by the absence of IgA plasma cells in the bone marrow, their reductions in the spleen, the blood and the lamina propria of the colon and the small intestine, concomitant with their accumulation and retention in mesenteric lymph nodes and Peyer’s patches. Most intriguingly, secretory IgA in the intestinal lumen was almost absent, dimeric serum IgA was drastically reduced and antigen-specific IgA responses to soluble *Salmonella* flagellin were blunted in KLF2-deficient mice. Perturbance of IgA plasma cell localization was caused by deregulation of CCR9, Integrin chains αM, α4, β7, and sphingosine-1-phosphate receptors. Hence, KLF2 not only orchestrates the localization of IgA plasma cells by fine-tuning chemokine receptors and adhesion molecules but also controls IgA responses to *Salmonella* flagellin.

## Introduction

Krüppel-like factor 2 (KLF2), a zinc-finger transcription factor, is a crucial regulator of differentiation, proliferation and activation of various cell types, including T- and B-lymphocytes^1–3^. Within the B cell lineage, KLF2 expression is induced during early B cell development in the bone marrow (BM) by signals of the pre-B cell receptor and maintained in naive, follicular B cells as well as in B1 cells^4–9^. Upon stimulation with antigens or mitogens, KLF2 is downregulated and re-expressed in memory B cells^5,6,10–13^. Previously, we showed that B cell-specific deletion of KLF2 resulted in profound changes in B cell homeostasis. Non-immunized KLF2-deficient mice displayed an expansion of follicular and mainly marginal zone B cells in the spleen. In addition, B1 cells in the peritoneum were undetectable using common B1 cell markers (such as CD5) and functionally altered^4–6^. Upon immunization with TNP-KLH, antigen-specific IgG-secreting plasma cells (PC) were virtually absent in the BM. Furthermore, we previously reported that serum IgA as well as the numbers and cellularity of Peyer’s patches (PP) in the gut were reduced in non-immunized KLF2-deficient mice^6^. This indicates a specific role for KLF2 in gut-associated lymphoid tissues (GALT) for IgA production and generation as well as maintenance of IgA^+^ PC.

IgA is the most abundantly produced IgH isotype in the human body^14^. Serum IgA is monomeric whereas secretory IgA (SIgA) in mucosal tissues is dimeric, with two IgA molecules connected through the J-chain^15–19^. Generation of class-switched IgA^+^ PC can be achieved in a T cell-dependent or T cell-independent manner and is triggered by cytokines, such as TGF−β^20,21^. The primary function of SIgA is to coat bacteria on mucosal surfaces to prevent bacteria from adhering and penetrating the epithelium^21^. The importance of IgA has been demonstrated in patients with a selective serum IgA deficiency, the most common primary immunodeficiency in humans. Although most of the IgA-deficient individuals are asymptomatic, some develop autoimmune symptoms, recurrent respiratory as well as gastrointestinal infections/disorders^22–24^. The molecular players that contribute to selective IgA deficiency are not well understood. Reduced serum IgA in KLF2-deficient mice suggested that KLF2 could be one factor that controls the development of IgA-producing PC. To test this hypothesis, we specifically analyzed the functional role of KLF2 in IgA^+^ PC generation, differentiation and maintenance, as well as its impact on IgA-mediated immune responses. Using KLF2:GFP reporter mice, we found that KLF2 is expressed predominantly in early IgA^+^ plasmablasts (PB) in mesenteric lymph nodes (mLN). Moreover, KLF2 was highly abundant in IgM^+^ and IgA^+^ PB in the blood. Using mice with a conditional mb1-cre-mediated deletion of KLF2 in the B cell lineage (KLF2 cKO mice), we demonstrated that IgA^+^ PC are virtually absent in the BM, reduced in the spleen, the blood, the small intestine (SI) and colonic lamina propria (LP), but accumulate in SI and colonic mLN as well as in the PP of KLF2 cKO mice. Absence of IgA^+^ PB/PC in the BM was not caused by defective BM entry but by defective exit from the mLN. Accordingly, we identified KLF2-regulated genes involved in adhesion and migration in mLN IgA^+^ PC, including CCR9, Integrins α4, αM, and β7, L-Selectin and sphingosine-1-phosphate receptors (S1PR) 1 and 4. Serum as well as intestinal and fecal IgA titers were significantly reduced, concomitant with the reduction of SI and colonic IgA^+^ PB/PC in KLF2 cKO mice. Moreover, KLF2-deficient animals were not able to mount antigen-specific IgA responses upon immunization with soluble flagellin (sFliC) from *Salmonella* Typhimurium. Hence, we identified KLF2 as a crucial factor that controls IgA^+^ PB/PC localization and IgA antibody responses.

## Results

### KLF2 is predominantly expressed in early IgA^+^ plasmablasts in mesenteric lymph nodes

**Fig. 1:**
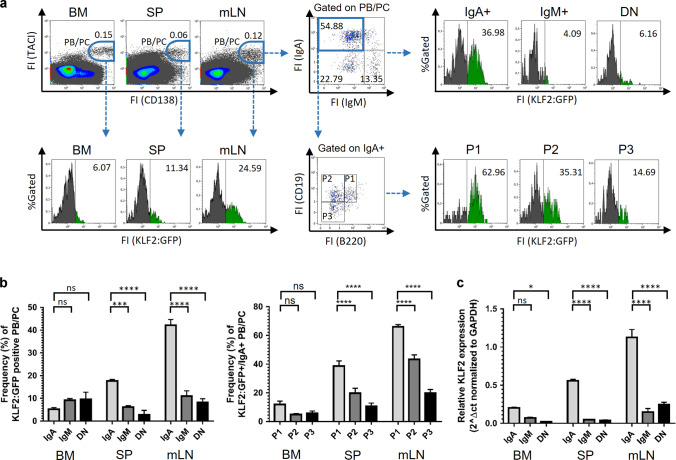
KLF2 is predominantly expressed in early IgA-positive plasmablasts in mesenteric lymph nodes. **a** Flow cytometric analyses of the frequencies of KLF2:GFP-positive cells within gated TACI^+^/CD138^+^ PB/PC in BM, spleen and mLN (left panel), within gated TACI^+^/CD138^+^/IgA^+^, TACI^+^/CD138^+^/IgM^+^ and TACI^+^/CD138^+^/IgA^−^/IgM^−^ (DN) PB/PC (upper right panel) as well as in PB/PC fractions P1 (TACI^+^/CD138^+^/CD19^+^/B220^+^), P2 (TACI^+^/CD138^+^/CD19^+^/B220^neg^) and P3 (TACI^+^/CD138^+^/CD19^int/neg^/B220^neg^) (lower right panel). Numbers indicate the percentages of cells in the respective gates. **b** Bar charts to the left represent the arithmetic mean values ± SEM of frequencies of KLF2:GFP-expressing cells within IgA^+^, IgM^+^ and DN PB/PC subsets in BM, spleen (SP) and mLN from *n* = 5 mice. Bar charts to the right represent the arithmetic mean values mice with SEM of frequencies of KLF2:GFP-expressing cells within IgA^+^ P1, P2 and P3 PB/PC subsets in BM, spleen and mLN from *n* = 5 mice. **c** TaqMan-PCR analyses of KLF2 transcripts of sorter-purified PC derived from BM, SP and mLN of C57BL/6 mice. Bar charts represent the arithmetic mean values of KLF2 RNA abundances in TACI^+^/CD138^+^ IgA^+^ or IgM^+^ or DN sorted PB/PC from the BM, SP or mLN of C57BL/6 mice normalized to GAPDH expression. Statistical analyses in **b** and **c** were performed for  PC subset or isotype comparison by two-way ANOVA with Sidak’s correction for multiple comparisons. FI Fluorescence intensity, FS Forward Scatter, SS Sideward Scatter, BM bone marrow, SP spleen, mLN mesenteric lymph nodes, FO B follicular B cells, MZ B Marginal Zone B cells, ns non-significant; *p < 0.05; ***p < 0.005; ****p < 0.001.

To analyze the abundance of KLF2 during PC differentiation, we performed flow cytometric analyses of PB/PC subsets in the spleen, BM, mLN and blood from reporter mice that express a KLF2-GFP fusion protein from the endogenous KLF2 promoter (KLF2:GFP mice^25^). First, we analyzed KLF2:GFP expression in various CD138^+^/TACI^+^ PB/PC subsets in BM, spleen, mLN, PP and peripheral blood^26,27^. We found the highest frequencies of KLF2:GFP-positive cells (~25%) within the TACI^+^/CD138^+^ PB/PC population in mLN (Fig. 1a). In the spleen, ~11% of the TACI^+^/CD138^+^ PB/PC were positive for KLF2, whereas in the BM, TACI^+^/CD138^+^/KLF2:GFP-positive cells were almost absent (Fig. 1b). Furthermore, we determined whether KLF2 is differentially expressed in PC expressing different IgH isotypes. We and others have shown that IgM^+^ and IgA^+^ PC, in contrast to IgG^+^ PC, still express a surface BCR^26,28–31^. Therefore, we analyzed KLF2:GFP expression in the mLN in surface IgA^+^, surface IgM^+^ and surface IgA^−^ and IgM^−^ (DN) TACI^+^/CD138^+^ PB/PC. The highest frequencies of KLF2:GFP-positive cells were found in the IgA^+^ PB/PC compartment (~37%, Fig. 1a, b). KLF2:GFP-positive cells could only be detected with very low frequencies in surface IgM^+^ and surface IgA^−^/IgM^−^ DN PB/PC (4% and 6%, respectively; Fig. 1a, b).

To investigate the abundance of KLF2 during IgA^+^ PC differentiation, we measured KLF2:GFP expression in early PB (P1) and early as well as late PC (P2 and P3, respectively) based on their differential expression of CD138, TACI, B220 and CD19^26,27,29^. In the mLN, KLF2-positive cells are enriched in the IgA^+^ PB P1 fraction (TACI^+^/CD138^+/^B220^+^/CD19^+^) with more than 60% KLF2:GFP-positive cells (Fig. 1a, b). However, the frequencies decreased from the early CD19^+^ P2 to the late CD19^−^ P3 compartment from ~40 to 17% KLF2:GFP-positive cells (Fig. 1b). These findings indicate that KLF2 expression follows or guides the maturation status of IgA^+^ PB. Of note, high frequencies of KLF2:GFP-positive cells were detectable within the IgA^+^ as well as the IgM^+^ TACI^+^/CD138^+^ PB subsets in the blood, suggesting that KLF2 plays a functional role at the migratory blood PB stage (Fig. S1A). Our observation that KLF2 is expressed in blood PB is in accordance with previously published KLF2 mRNA analysis^32^. KLF2:GFP expression in PB/PC subsets in the PP, however, was virtually absent (Fig. S1A). To validate these findings, we sorter-purified IgM^+^ as well as IgA^+^ PB/PC from lymphatic tissues of WT C57Bl/6 mice (sorting strategy depicted in Fig. S1B) and determined KLF2 transcript abundances by quantitative TaqMan-PCR. As shown in Fig. 1c and in accordance with our KLF2:GFP data, we found the highest abundance of KLF2 mRNA in IgA^+^ PB/PC from the mLN while KLF2 transcripts were barely detectable in PC in the spleen and BM.

### IgA-producing plasma cells are absent in the BM and accumulate in the mesenteric lymph nodes of KLF2-deficient mice

**Fig. 2:**
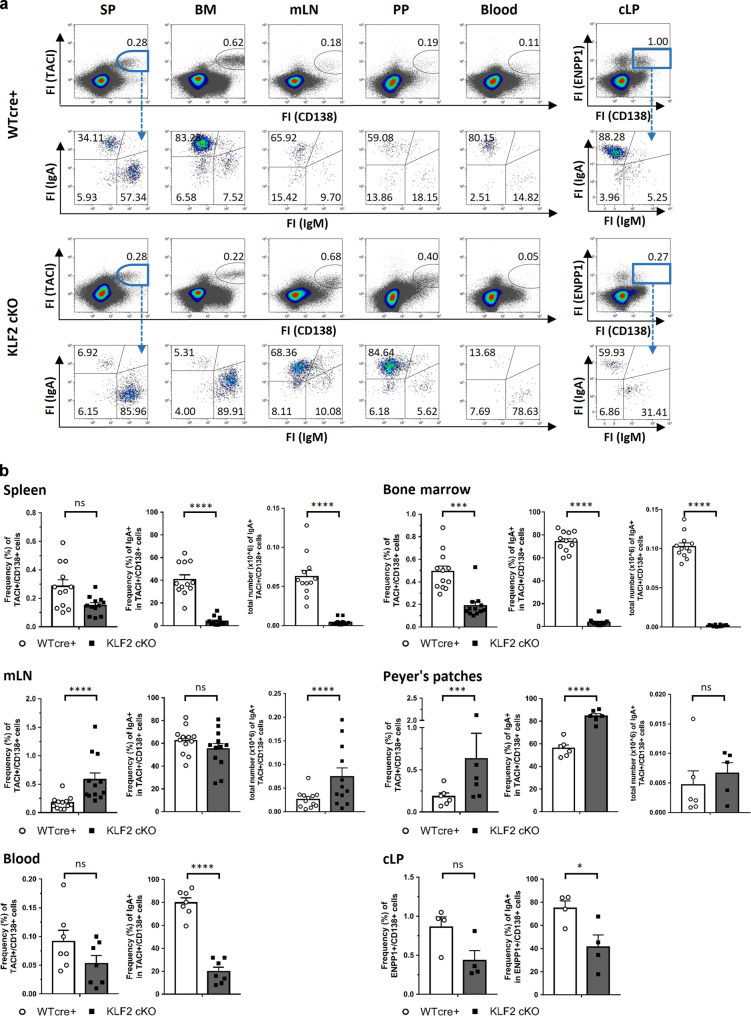
IgA-producing plasma cells are absent in the BM and accumulate in the mesenteric lymph nodes of KLF2-deficient mice. **a** Flow cytometric analysis of PB/PC defined as CD138^+^/TACI^+^ for SP, BM, Blood, mLN and PP or CD138^+^/ENPP1^+^ for colon LP (cLP) divided into IgA^+^, IgM^+^ and IgA^−^/IgM^−^ PB/PC isotypes of of WT cre+ control (upper plots) and KLF2 cKO (lower plots) mice; numbers indicate the percentage of cells in the respective gates; **b** bar charts show the arithmetic mean with SEM of CD138^+^/TACI^+^ PB/PC frequencies (left diagram), frequencies of TACI^+^/CD138^+^/IgA^+^ (mid diagram) and total cell numbers of TACI^+^/CD138^+^/IgA^+^ (right diagram) from SP, BM, mLN, PP and the blood from WT cre+ mice (white circles, white bars) and KLF2 cKO mice (black squares, gray bars); *N* = 2 experiments with *n* = 6–7 (blood and PP) and *n* = 12 (SP, BM, mLN) mice; statistical analyses were performed for genotype comparison by unpaired *t*-test. For cLP: bar charts show the arithmetic mean with SEM of CD138^+^/ENPP1^+^ PB/PC frequencies (left diagram) and frequencies of IgA^+^ PB/PC (right diagram) of KLF2 cKO (gray bars, black squares) and WT cre+ control (white bars, white circles) mice; *n* = 4; statistics were calculated by using an unpaired *t*-test. FI fluorescence intensity, BM bone marrow, SP spleen, mLN mesenteric lymph nodes, PP Peyer’s patches, cLP colon lamina propria, ns non-significant; *p < 0.05; **p < 0.01; ***p < 0.005; ****p < 0.001.

To determine the functional role of KLF2 in PC generation and maintenance, we analyzed PB/PC subsets in lymphatic organs from mice with a conditional B cell-specific deletion of KLF2 (KLF2 cKO). These mice were established by crossing the mb1-cre deleter strain to a mouse line with floxed KLF2 alleles^6^. Mb1-cre mice with wildtype KLF2 alleles (WT cre+) served as controls. Flow cytometric analyses revealed that frequencies and absolute numbers of TACI^+^/CD138^+^ PB/PC were ~3-fold decreased in the BM, but significantly increased (~3-fold) in the mLN (Fig. 2a, b) of KLF2 cKO mice. Specifically, surface IgA^+^ TACI^+^/CD138^+^ PB/PC were significantly reduced in BM, spleen and blood, indicating a specific impact of KLF2-deficiency on the homeostasis of IgA^+^ PB/PC. In contrast, we found a significant increase in the frequencies of IgA^+^ PB/PC in the PP. However, due to the fact that the number of PP are decreased in KLF2 cKO mice^6^, the total numbers of IgA^+^ PB/PC remained unaltered (Fig. 2a, b). Strikingly, we found significantly higher numbers of TACI^+^/CD138^+^/IgA^+^ PB/PC in the mLN of KLF2 cKO compared to WT cre+ control animals, indicating that KLF2 plays a role in either the generation of IgA^+^ PB or in the exit of IgA^+^ PB from the mLN (Fig. 2b). Moreover, we observed a significant increase in the P2 subset in the mLN (Fig. S2A).

**Fig. 3:**
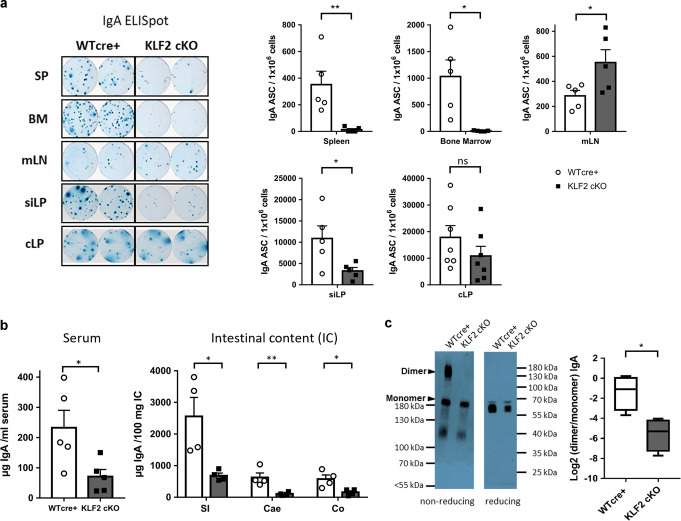
Reduction of IgA-secreting plasma cells in the lamina propria of the gut affects the abundance of secreted luminal IgA in KLF2-deficient mice. **a** Representative ELISpots and statistical analysis of SP, BM, mLN, siLP and cLP IgA-secreting cells of KLF2 cKO (gray bars, black squares) and WT cre+ control (white bars, white circles) mice; *n* = 5–7 mice, statistics were calculated by using an unpaired *t*-test. **b** ELISA analysis of serum IgA concentrations (left), mean with SEM, *n* = 5 mice; and ELISA analysis of secreted IgA (SIgA) in intestinal content (IC) of the small intestine (SI), Cecum (Cae) and Colon (CO) (right) of KLF2 cKO (gray bars, black squares) and WT cre+ control (white bars, white circles) mice, mean concentrations with SEM, *n* = 4; statistics were calculated with unpaired *t*-test. **c** Non-reducing and reducing western blot analysis of natural monomeric and dimeric IgA in the serum of WT cre+ control and KLF2 cKO mice. Non-reducing conditions (left): monomeric IgA (~180 kDa) and dimeric IgA (~360 kDa). Reducing conditions (right): IgA (α heavy chain: ~60 kDa). Box plot diagram shows Log2 dimer to monomer ratio of serum IgA in WT cre+ controls (white box plot) and KLF2 cKO (gray box plot) mice. The relative amount of IgA was determined by analysis of the “area under the curve” in ImageJ using the “gel analysis” tool. Box plots show arithmetic mean ± SEM, *n* = 4 mice; unpaired *t*-test. BM bone marrow, SP spleen, mLN mesenteric lymph nodes, siLP small intestine lamina propria, cLP colon lamina propria, IC intestinal content, ns non-significant; *p < 0.05; **p < 0.01.

According to their anatomical location and immunological function, the draining mLN can be subdivided into mLN draining the small intestine (si-mLN) and mLN surveilling the colon (c-mLN)^33^. To determine whether deletion of KLF2 affects the IgA^+^ PB/PC populations in the si-mLN and the c-mLN, we quantified IgA^+^ PB/PC by flow cytometric analyses based on CD138/TACI/IgA and J-chain stainings. In accordance with our previous findings using total mLN cells, IgA^+^ PB/PC accumulated in both, the si-mLN and the c-mLN (Fig. S2C). Moreover, we determined the frequencies IgA^+^ PB/PC in the colonic LP (cLP) of the gut. Due to the enzymatic digestion which is necessary for isolation of LP cells, the protease-sensitive marker TACI could not be used to identify PC. Instead, we used ectonucleotide pyrophosphatase/phosphodiesterase 1 (ENPP1) as a PB/PC marker^27,34^. Flow cytometric analyses identified less CD138^+^/ENPP1^+^ PB/PC in the cLP of KLF2 cKO mice and, more importantly, significant lower frequencies of CD138^+^/ENPP1^+^/IgA^+^ PB/PC (~75% IgA^+^ within the CD138^+^/ENPP1^+^ population in WT cre+ and ~41% in KLF2 cKO, respectively, Fig. 2a, b). No changes in the frequencies in B220^+^/CD19^+^, B220^−^/CD19^+^ and B220^−^/CD19^−^ subsets of CD138^+^/ENPP1^+^ PB/PC were observed (Fig. S2B). To determine whether deletion of KLF2 affects IgA^+^ PB/PC in the small intestine lamina propria (siLP), we quantified IgA-secreting PB/PC by ELISpot. As depicted in Fig. 3a, we found a significant reduction of IgA-secreting PB/PC in the siLP of KLF2 cKO mice. In support, histological analyses using anti-IgA and anti-J-chain co-stainings to distinguish IgA^+^/J-chain^+^ PB/PC from IgA^+^/J-chain^−^ memory B cells revealed a reduction of IgA^+^/J-chain^+^ PB/PC in both, the SI and the colon (CO) of KLF2 cKO mice (Fig. S3A). ELISpot analyses confirmed the absence of IgA-secreting cells in the BM and the spleen, as well as their accumulation in the mLN of KLF2 cKO mice (Fig. 3a).

Next, we assessed the effect of KLF2-deficiency on serum and fecal IgA antibody titers by ELISA. As depicted in Fig. 3b, serum IgA is strongly reduced, a finding that supports previous observations^5,6^. Additionally, we found a striking reduction of fecal IgA in KLF2 cKO mice (Fig. S3B). To corroborate these findings, we determined the IgA amounts in the intestinal luminal content of the SI, the cecum (Cae) and the CO by ELISA. We found a significant reduction of SIgA in all analyzed segments of the intestine (Fig. 3b). In addition, IgA staining of bacteria in the SI, the Cae and the CO revealed significant reductions of IgA labeling of bacteria in the Cae and the CO (Fig. S3C). Hence, deletion of KLF2 resulted in the accumulation of IgA^+^ PB/PC in the mLN and the PP, concomitant with a reduction of IgA^+^ PB/PC in the spleen and the blood, and their absence in the BM. Moreover, IgA^+^ PB/PC numbers were reduced in the LP of the CO and the SI. In support of these findings, a severe reduction of SIgA in all parts of the intestine was observed in the absence of KLF2. In this context, we analyzed serum samples from WT cre+ and KLF2 cKO mice for the presence of monomeric and dimeric IgA by non-reducing polyacrylamide gel electrophoresis with subsequent western blotting. As shown in Fig. 3c, we found a significant reduction of dimeric IgA in the serum of KLF2 cKO, indicating that the production of dimeric IgA is perturbed in KLF2 cKO mice, which is in line with the observed reduction of IgA^+^ PB/PC in the LP of the SI and the CO.

### Loss of KLF2 results in impaired integrin and chemokine receptor expression on IgA plasmablasts in mesenteric lymph nodes

To determine how KLF2-defiency results in IgA^+^ PB/PC accumulation in the mLN on a molecular level, we analyzed the transcriptome of purified IgA^+^ PB (TACI^+^/CD138^+^/B220^+^/IgA^+^) from the mLN of KLF2 cKO and WT cre+ control mice (sorting strategy and gating depicted in Fig. S4A). In summary, we detected 192 genes that were significantly upregulated (log2FC > 1, FDR ≤ 0.05, depicted in red), 571 significantly downregulated genes (log2FC < −1 1, FDR ≤ 0.05, depicted in blue) as well as 11825 genes showing non-significant changes (depicted in black) between WT cre+ control and KLF2 cKO IgA^+^ PB, depicted as a Volcano Plot in Fig. S4B. To verify whether KLF2 affects PB/PC generation and differentiation, we analyzed transcripts of genes known to be critical for PC development and maintenance (Fig. S4C). Transcript abundances of the key regulators of PC differentiation Blimp1 (*Prdm1*), IRF-4 and Xbp-1 were not affected by KLF2 deletion (Fig. S4C). Thus, we conclude that PB/PC differentiation per se is not disturbed by the loss of KLF2.

**Fig. 4:**
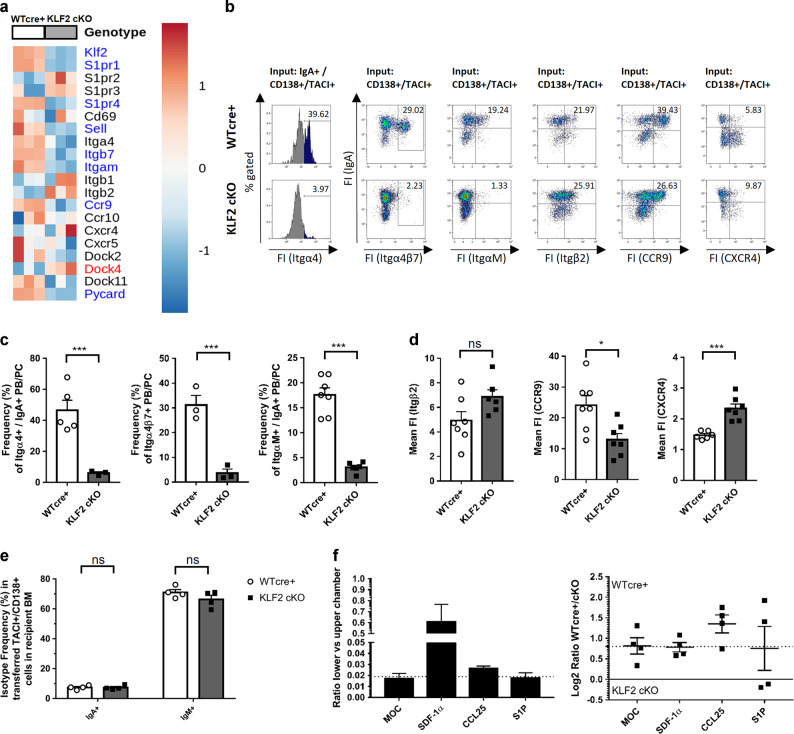
Loss of KLF2 results in impaired integrin and chemokine receptor expression on IgA plasmablasts in mesenteric lymph nodes. **a** Heat map of selected genes involved in migration and cell adhesion identified by RNA-seq analysis in TACI^+^/CD138^+^/IgA^+^/B220^+^ sorter-purified PB from the mLN of KLF2 cKO and WT cre+ control mice. Genes highlighted in blue were significantly downregulated (log2FC < −1, FDR ≤ 0.05), genes highlighted in red were significantly upregulated (log2FC > 1, FDR ≤ 0.05). **b** Flow cytometric analysis of Integrin α4 (CD49d), Integrin α4β7, Integrin αΜ (CD11b), Integrin β2 (CD18), CCR9 and CXCR4 surface expression on WT cre+ and KLF2 cKO TACI^+^/CD138^+^/IgA^+^ PB/PC in the mLN. **c** Bar charts show mean percentages with SEM of TACI^+^/CD138^+^/IgA^+^/Itgα4^+^ PB/PC (left), TACI^+^/CD138^+^/Itgβ7^+^ PB/PC (middle) and TACI^+^/CD138^+^/ItgαM^+^ PB/PC (right) from WT cre+ (open circles, white bars) or KLF2 cKO (black squares, gray bars) mice in the mLN. For Itgα4 and Itgα4β7: *n* = 3–5 mice, unpaired *t*-test; For ItgαM: *n* = 6–7 mice, statistic was performed using Mann–Whitney *U* test. **d** Bar charts show mean fluorescent intensities ± SEM of TACI^+^/CD138^+^/IgA^+^/Itgβ2^+^ PB/PC (left), TACI^+^/CD138^+^/IgA^+^/CCR9^+^ PB/PC (middle) and TACI^+^/CD138^+^/IgA^+^/CXCR4^+^ PB/PC (right) from WT cre+ (open circles, white bars) or KLF2 cKO (black squares, gray bars) mice in the mLN. *n* = 6–7 mice, Mann–Whitney *U* test. **e** Bar charts show the arithmetic mean with SEM of CD138^+^/TACI^+^/IgA^+^ and CD138^+^/TACI^+^/IgM^+^ IgA switch mix activated cells after 3 days of competitive transfer into Rag^−/−^ mice in the BM; in vitro activation of splenic B cells was assessed on day 3 by flow cytometry; equal living cell numbers of cultured KLF2 cKO and WT cre+ cells were reciprocally labeled with either eFluor670 or eFluor450 proliferation dyes and mixed before intravenous injection into recipient mice; *n* = 4 mice, statistics were performed using unpaired *t*-test. **f** Migratory activity of CD138^+^/TACI^+^ IgA^+^ cells in transwell assays (3 h). Left diagram shows the basic migratory activity of CD138^+^/TACI^+^ activated cells in medium only control (MOC) and toward SDF-1α (100 nM), CCL25 (300 nM) and S1P (100 nM) in the lower chamber, calculated by CD138^+^/TACI^+^ cell number (lower chamber) divided by CD138^+^/TACI^+^ cell number (upper chamber). Motility baseline of non-directed migration in MOC is indicated by the dashed line. Bars indicate mean value ± SEM, all values above baseline motility indicate active migration toward the indicated chemokine. Right diagram shows the mean Log2 ratio ± SEM of CD138^+^/TACI^+^ KLF2 cKO and WT cre+ cells, calculated by Log2 ((lower/upper WT cre+)/(lower/upper KLF2 cKO)) cell number. Equal migration of WT cre+ and KLF2 cKO cells results in a value of zero (black line), baseline migration of MOC only control results in a positive value, indicating a higher motility of WT cre+ cells, baseline (non-directed) motility is indicated by dashed line, deviations from this line indicate advantageous WT cre+ cells motility (above dashed line) or advantageous KLF2 cKO motility (below dashed line) directed toward the indicated chemokine. FS Forward Scatter, SS Side Scatter, FI fluorescence intensity, mLN mesenteric lymph nodes, ns non-significant; *p < 0.05; ***p < 0.005.

The top 50 differentially expressed genes (Fig. S4D) included factors involved in migration and adhesion such as CCR9, ItgαM, Itgβ7 as well as members of the S1PR family, all of which were significantly downregulated in KLF2 cKO IgA^+^ PB (Fig. 4a). As KLF2 regulates L-Selectin and Itgβ7 expression in T and B cells as well as S1PR1 in T cells^3,5,6,35,36^, we analyzed RNA abundances of migration and adhesion factors as well as chemokine receptors in more detail. As depicted in Fig. 4a, CCR9, an important chemokine receptor for homing of lymphocytes to the LP^37^, is downregulated in KLF2 cKO IgA^+^ PB. Moreover, ItgαM RNA abundance was significantly downregulated in KLF2 cKO IgA^+^ PB. The ItgαM chain (CD11b) pairs with the Itgβ2 chain to form a cell surface complex involved in cell migration^38^. Itgβ2 plays a functional role in the exit of newly generated PCs from lymph nodes (LN) as PC interact with their surface Itgβ2 to ICAM-1-expressing cells in the medullary cord of LN. Most importantly, in Itgβ2-deficient mice, PC accumulated in LN and were absent in the BM^39^. As depicted in Fig. 4a, members of the S1PR family were differentially regulated. S1PR-mediated chemotaxis to the ligand S1P attracts lymphocytes from tissues to the blood, where high concentrations of S1P are found^40^. S1PR1 and S1PR4 were significantly downregulated on RNA levels in KLF2 cKO IgA^+^ PB, whereas S1PR2 and S1PR3 expression was not significantly altered. L-Selectin and Itgβ7 were, as expected, downregulated in IgA^+^ KLF2 cKO PB. As Itgα4 chain is the critical component of Itgα4β1 and Itgα4β7 complexes that are crucial for PC/stromal cell interaction in the PC survival niche^27,41^, we analyzed Itgα4 as well as Itgβ7 protein expression on the surface of PB/PC by flow cytometry. Indeed, we found that frequencies of Itgα4^+^, Itgβ7^+^ as well as ItgαM^+^ IgA^+^ PB/PC were significantly reduced in KLF2 cKO mLN (Fig. 4b, c). However, surface expression of Itgβ2 was not altered. Moreover, we found a reduced surface abundance of CCR9 on KLF2 cKO IgA^+^ PB. CXCR4 surface abundance, however, was higher on KLF2 cKO IgA^+^ PB/PC (Fig. 4b, d). However, the abundances of CXCR4 on the surfaces of WT cre+ and KLF2 cKO IgA^+^ PC was in general significantly lower compared to WT cre+ and KLF2 cKO IgM^+^ PC (Fig. S4E).

As we found a profound change in the abundances of chemokine receptors and adhesion molecules, we asked whether the observed absence of IgA^+^ PC in the BM was due to impaired BM homing/BM entry. To address this question, we performed adoptive transfer experiments of in vitro generated, class-switched IgA^+^ PB derived from either KLF2 cKO or WT cre+ control mice (stimulated with cytokine switch mix, experimental setup and cell culture analysis see Fig. S4F, G) into Rag^−/−^ recipient mice. in vitro stimulated KLF2 cKO and WT cre+ control cells expressed the PC markers CD138 and TACI and contained IgA^+^, IgM^+^ as well as IgA^−^/IgM^−^ PB. TACI and CD138 expression as well as viabilities of KLF2 cKO and WT cre+ control cultures were comparable on day 3 after stimulation. Surface abundances of CCR9 and Itgα4β7 were significantly lower on in vitro-activated TACI^+^/CD138^+^ KLF2 cKO cells (Fig. S4G) in accordance with our in vivo observations. On day 3, KLF2 cKO and WT cre+ control B cells were reciprocally labeled with proliferation dyes eFlour670 and eFlour450 to exclude adverse effects caused by the fluorescent dyes. Equal numbers of KLF2 cKO and WT cre+ control cells were mixed and transferred into Rag^−/−^ mice by intravenous (i.v.) injection. Three days after injection, we determined the frequencies of transferred TACI^+^/CD138^+^ PB/PC in BM, spleen and blood as well as mLN and cLP in the recipients by flow cytometry. Transferred activated TACI^+^/CD138^neg^ B cells accumulated in the blood, while almost no labeled cells were detectable in the mLN and cLP (Fig. S4F). As shown in Fig. S4F, similar frequencies of transferred KLF2-deficient TACI^+^/CD138^+^ PB/PC compared to the WT cre+ cells were detectable in the BM and the spleen of recipient mice. Most importantly, KLF2-deficient IgA^+^ and IgM^+ −^ PB/PC migrated to the BM with similar efficiencies compared to the WT cre+ control cells (Fig. 4e). Hence, we conclude that homing of IgA^+^ PB to the BM can occur even in the absence of KLF2.

To determine the functional impact of CCR9 and S1PR dysregulation on the migration ability of KLF2 cKO PB, we performed transwell assays using in vitro-activated WT cre+ and KLF2 cKO PB in response to CCL25 and S1P (Fig. S4H). SDF-1α served as a positive control. As shown in Fig. 4f, KLF2 cKO PB showed a decreased basal motility and a decrease of CCR9-dependent migration to a CCL25 gradient. S1P-dependent migration was not detectable for WT cre+ and KLF2 cKO cells. These findings demonstrate that KLF2 deletion resulted in an impairment of CCR9-mediated chemotaxis to CCL25 gradients which very likely contributes to the reduced numbers of IgA^+^ PB/PC observed in the CO and the SI of KLF2 cKO mice.

### KLF2-deficient animals fail to mount an antigen-specific IgA immune responses to *Salmonella* Typhimurium antigen flagellin (sFliC)

**Fig. 5:**
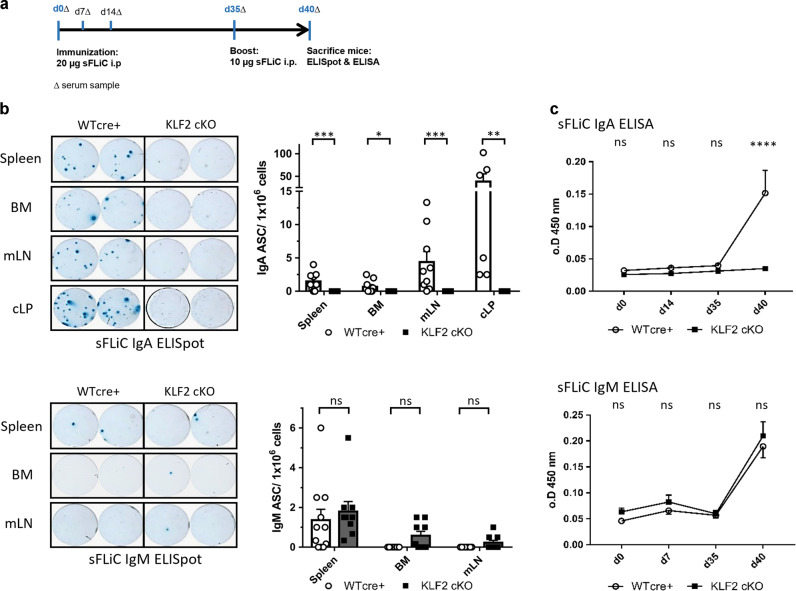
KLF2-deficient animals fail to mount an antigen-specific IgA immune response to *Salmonella* Typhimurium antigen Flagellin (sFliC) and show impaired IgA dimerization. **a** Schematic overview of the immunization workflow; serum samples (Δ) were collected on day 0 before immunization, day 7, 14 and 35 before boost immunization, as well as on day 40. **b** Representative ELISpots of one given cell dilution (left) and statistics (right) of the arithmetic mean with SEM of ELISpot analysis for anti-sFliC IgA (upper) and IgM (lower) antibody-secreting cells (ASC) in SP, BM, mLN and colon LP (cLP) of KLF2 cKO (gray bars, black squares) and WT cre+ control (white bars, white circles) mice. *N* = 3 *n* = 9–10 for BM, SP, mLN and *n* = 6 for cLP, removal of definitive outliers by ROUT (*Q* = 0.1%) test, statistics was performed applying a Mann–Whitney *U* test; **c** Arithmetic means with SEM of relative optical densities (o.D.) of sFliC-specific serum IgA (upper) and IgM (lower) at indicated time points after immunization of KLF2 cKO (black squares) and WT cre+ control (white circles) mice; *n* = 5, statistical analyses were performed for time point and genotype comparison by two-way ANOVA with Sidak’s correction for multiple comparisons. PB plasmablast, PC plasma cell, BM bone marrow, mLN mesenteric lymph nodes, cLP colon lamina propria, sFliC soluble recombinant Flagellin antigen, ASC antibody-secreting cells, o.D. optical density, ns non-significant; *p < 0.05; ***p < 0.005; ****p < 0.001.

To determine whether KLF2-deficiency affects IgA immune responses, we immunized KLF2 cKO and WT cre+ control mice intraperitoneally with soluble flagellin (sFliC) from *Salmonella* Typhimurium and monitored antigen-specific immune responses by ELISpot and ELISA. sFliC has been shown to induce local IgA responses in the mLN as well as a systemic IgG response with sFLIC-specific IgG PC detectable in the spleen^42–44^. sFliC-immunized KLF2 cKO and WT cre+ control mice were boosted on day 35 (Fig. 5a). Antigen-specific PB/PC numbers in BM, spleen, CO and mLN were enumerated by ELISpot analyses on day 5 after boost immunization. As depicted in Fig. 5b, we found that sFliC-specific IgA-secreting cells were absent in the spleen, the BM, the mLN and the LP of the CO of KLF2 cKO mice but could clearly be detected in immunized WT cre+ control mice. ELISA assays confirmed the absence of sFliC-specific serum IgA responses (Fig. 5c). In contrast, sFliC-specific IgM-secreting cells were unaltered in KLF2 cKO BM, spleen and mLN (Fig. 5b) and serum IgM and was unperturbed (Fig. 5c). In summary, KLF2 deletion blunts the generation of antigen-specific IgA responses upon sFliC immunization concomitant with the complete absence of antigen-specific PC in the BM, SP, mLN and colonic LP.

## Discussion

Previous studies revealed that KLF2 controls B and T cell pool sizes as well as their localization in the organism^3,5,6,45–50^. Here, we characterized KLF2 expression in different PB/PC subsets, determined the role of KLF2 for the localization of IgA^+^ PB/PC subsets in non-immunized mice, and investigated the impact of KLF2 deletion on antigen-specific IgA responses in sFLiC-immunized mice. Using KLF2:GFP reporter mice^25^, we revealed that KLF2 expression is highest within the early IgA^+^ P1 PB/PC subset indicating that KLF2 is of functional relevance early in IgA^+^ PC generation and development. Moreover, Hart et al. showed that B1 cells are characterized by high KLF2 expression^5^. As B1 cells essentially contribute to the pool of IgA^+^ PB/PC^51^, high KLF2 abundance in early IgA^+^ PB might be indicative of their B1 origin. Furthermore, in BM PB and PC subsets, KLF2 was only abundant at very low levels. However, we found a high proportion of KLF2-expressing PB in the blood regardless of their isotype. In support, KLF2 and S1PR1 RNA was shown to be upregulated in blood PB compared to splenic PB^32^. PB in the blood are exposed to the bloodstream and, therefore, shear forces might trigger the upregulation of KLF2, similar to the mechanisms observed in endothelial cells^52^.

In KLF2 cKO mice, IgA^+^ PC in the BM were virtually absent, and in the spleen and blood, the IgA^+^ PB/PC compartment was strongly reduced. We excluded a defect in BM homing/entry by transfer experiments that demonstrated similar BM entry abilities of KLF2-cKO IgA^+^ PB compared to controls. However, a significant accumulation of IgA^+^ PB/PC in the mLN as well as in the few remaining PP was observed. Based on their anatomical location and immunological functions, mLN can be subdivided in draining mLN of the SI and of the CO^33^. Our analysis revealed that accumulation of IgA^+^ PB/PC occurs in mLN of the SI as well as CO of KLF2 cKO mice. Thus, the loss of KLF2 resulted in retention and accumulation of IgA^+^ PB/PC in the PP and the draining mLN of SI and CO. Accumulation of IgA^+^ PB/PC in the mLN might be the consequence of impaired exit and fostered retention caused by dysregulation of adhesion molecules, chemokine receptors and migratory factors. In support, downregulation of KLF2 and its target gene S1PR1 is a common pathway to enable tissue residence of many other cell types, such as memory T cells^3^. Along this line, we found that S1PR1 and S1PR4 are downregulated in KLF2 cKO IgA^+^ PB. Moreover, ItgαM which interacts with the Itgβ2 chain to form a functional surface complex, is significantly downregulated in KLF2 cKO IgA^+^ PB^53^. In this context, Pabst et al. showed that lack of Itgβ2 resulted in the defective exit of PC from LN^39^. Hence, the observed decrease in ItgαM and in addition, the downregulation of S1PR1 might be causative for the accumulation of IgA^+^ PB in the mLN.

It has been shown that the IgA^+^ PC pool which is generated in mucosal tissues contributes to the BM IgA^+^ pool^54^. Our transfer experiments showed that KLF2-deficient PB are capable of BM entry when injected into the bloodstream. Even though some KLF2-deficient IgA^+^ PB might reach their niches in the BM, their ability to establish intimate contacts to stromal cells might be impaired due to the observed reduction of Itgα4 and as a consequence of Itgα4β1^27^.

Deletion of KLF2 resulted in the loss of Itgβ7 surface expression on IgA^+^ PB/PC, a crucially important integrin for homing to GALT structures^55^. Accordingly, we detected fewer IgA^+^ PB/PC in the SI and in the CO of KLF2 cKO mice. Itgβ7 can pair with Itgα4 and ItgαE chains to form the heterodimeric Itgα4β7 and ItgαEβ7 surface receptors. Itgα4β7 is crucial for the recruitment of immune cells to the LP, whereas ItgαEβ7 is expressed on a subset of IgA^+^ PC and establishes the contact with intestinal epithelial cells, thereby promoting transcytosis of IgA^55,56^. Thus, KLF2 controls Itgβ7-mediated recruitment of IgA^+^ PC to the LP and might be involved in ItgαEβ7-mediated interaction of IgA^+^ PC with the epithelium. The downregulation of Itgβ7 is implicated in diseases such as the Kabuki syndrome, which is caused by mutations in the gene for the histone-lysine N-methyltransferase KMT2D^57–59^. In a haplo-insufficient KMT2D^+/βGEO^ mouse model, reduced Itgβ7 expression resulted in abnormal PP size and numbers as well as impaired B and IgA^+^ PC populations, a phenotype similar to our B cell-specific KLF2 cKO mouse^5,6,60^. Both, the KMT2D^+/GEO^ and KLF2 cKO mouse models exhibit a dysregulation of Itgβ7 resulting in IgA deficiency. As KLF2 transcripts were not significantly affected in KMT2D-deficient B cells^61^, we assume that KMT2D and KLF2 regulate Itgβ7 expression independently.

CCR9, one of the key chemokine receptors mediating lymphocyte homing to the LP^37^, was significantly downregulated on KLF2–deficient IgA^+^ PB and migration of KLF2-deficient IgA^+^ PB to the CCR9 ligand CCL25 was impaired. Downregulation of CCR9, together with the observed lack of Itgβ7 might therefore be causative for the reduction of IgA^+^ PC observed in the colonic and the small intestinal LP of our KLF2 cKO mouse. The substantial reduction of SIgA in the lumen of the SI, the CO and the Cae as measured by intestinal content ELISA and by IgA-coating of bacteria along the gut of KLF2 cKO mice is concomitant with the observed reduction of IgA^+^ PB/PC in the LP of the SI and the CO. Along this line, we also found a significant decrease of dimeric IgA in the serum of KLF2 cKO mice.

To functionally assess the ability of KLF2 cKO to mount an antigen-specific IgA immune response, we performed immunization experiments with flagellin (sFliC), an immuno-dominant antigen from *Salmonella* Typhimurium. sFliC induces local IgA responses in the mLN and systemic IgG responses^42–44^. Our ELISpot analyses showed that sFliC-specific IgA-secreting cells were virtually absent in the spleen, the BM, the mLN and the cLP of KLF2 cKO mice concomitant with the absence of anti-sFliC-specific IgA antibodies in the serum of KLF2 cKO mice. Thus, we revealed that KLF2 cKO animals are not capable of mounting antigen-specific IgA immune responses to sFLiC protein.

In summary, B cell-specific deletion of KLF2 in mice resulted in accumulation of IgA^+^ PB/PC in the mLN and PP, their subsequent reductions in the spleen, the blood and the BM, and in perturbed IgA^+^ PB homing to the LP of the SI and the CO. KLF2 controls LN egress of IgA^+^ PB/PC through regulation of ItgαM and S1PRs, and mediates intestinal homing through upregulating Itgβ7 and CCR9 expression. Due to the impaired compartmentalization of IgA^+^ PB/PC, KLF2 cKO mice exhibit a severe reduction of serum IgA as well as intestinal SIgA concomitant with defective IgA responses to bacterial antigens, such as *Salmonella* flagellin.

## Material and methods

### Experimental model and subject details

#### Mouse models

KLF2 fl/fl mb1cre+/− (KLF2 cKO), KLF2 wt/wt mb1cre+/− (WT cre+), KLF2:GFP reporter mice (all on a C57BL/6 genetic background) and Rag-deficient (Rag^−/−^) mice were bred and kept in the Franz‐Penzoldt-Center and in the Nikolaus-Fiebiger-Center animal facility (University of Erlangen‐Nürnberg, Erlangen, Germany) under specific-pathogen‐free conditions. To reduce stress, all cages contained the following enrichment materials: cardboard/paper houses, cotton rolls and cotton “Nestlets” (Plexx, BV, the Netherlands). The health status of all mice was controlled daily by trained staff. All animal experiments were performed according to institutional and national guidelines (Permit Numbers: TS 04/07 and TS 05/07, Amt für Veterinärwesen und gesundheitlichen Verbraucherschutz der Stadt Erlangen, Erlangen, Germany; Az.55.2.2-2532-2-639, Az.55.2.2-2532-2-753, Regierung von Unterfranken, Würzburg, Germany). All experiments were carried out by trained staff using established protocols. For immunization experiments, co-housed, male and female mice with an age between 10–20 weeks at the date of immunization were used. For all other experiments, co-housed and age-matched, male and female mice with an age older than 8 weeks were used. To obtain littermate controls, KLF2 fl/wt mb1cre+/− males were bred with KLF2 fl/wt mb1cre+/− females resulting in KLF2 fl/fl mb1cre+/− and KLF2 wt/wt mb1cre+/− offspring^6^. KLF2:GFP reporter mice^25^ were kindly provided by Andreas Hutloff (University Hospital Schleswig-Holstein, Kiel). C57BL/6 mice were obtained from Janvier Labs (Le Genest-Saint-Isle, France) and kept at the Nikolaus-Fiebiger-Center animal facility.

### Method details

#### Preparation of single-cell suspension of murine tissues for flow cytometric and ELISpot analyses

For flow cytometric analysis, BM, spleen (SP), blood (350–500 µl), PP, total mLN and colon lamina propria (cLP) tissue were analyzed. SI draining mLN and CO draining mLN were isolated according to Houston et al.^33^. For BM, one or two femora were flushed with cold R10 medium; SP, mLN and PP were homogenized using 70 µm cell strainers with 5 ml cold R10 medium; Blood was collected in tubes containing 50 µl 0.5 M EDTA (Invitrogen; 15575-038); cLP cells were purified by DNAse/Collagenase digestion (Miltenyi; protocol see below). For BM, SP and Blood, erythrocyte lysis was performed for 5 min with 5 ml red blood cell lysis buffer (Biolegend; 420301) at RT, stopped by the addition of 5 ml cold R10 media. Before antibody staining, cells were resuspended in 3–10 ml R10 media, filtered through 30 µm cell filter and living cell numbers (CN) were determined by using the NC3000 nucleocounter cell count and viability assay (Chemometec, protocol see below).

#### Lamina propria single-cell preparation for flow cytometric analysis and ELISpot

For LP single-cell suspensions, up to 1 g of gut tissue was cleaned from fatty tissue and flushed with cold PBS + 2% BSA. Tissue was opened and cleaned from feces and mucus with cold PBS + 2% BSA in four washing steps. Tissue was cut into 1 cm pieces and pre-digested two times for 15 min in R10 medium/5 mM EDTA at 37 °C in a water bath. After pre-digestion incubation steps, tissue was mixed vigorously by hand to get rid of intra-epithelial cells. Pre-digested tissue was washed in cold PBS + 2% BSA and cut into small pieces using a scalpel. Tissue was digested for 22 min in 10 ml RPMI1640 + Miltenyi Lamina Propria Dissociation Kit, mouse enzymes (100 µl D, 50 µl R, 12.5 µl A; Miltenyi; 130-097-410) at 37 °C while shaking. Tissue was washed with cold R10 and filtered through 70 µm filters. Cell samples were centrifuged in 20% Percoll (GE Healthcare; 17-0891-02) in R10 suspensions for 20 min at 2200 rpm (without brake). Single cells were resuspended in 2 ml R10 media for analysis.

#### Cell count and viability assay with NC3000 nucleocounter

For cell count and viability measurements, 38 µl of single-cell suspension was mixed with 2 µl of Chemometec Solution 13 (DAPI and Acridine Orange; Chemometec; 910-3013). In total, 10 µl of stained cells were placed on an 8-chamber slide and cell- and viability count was performed on an NC3000 nucleocounter (Chemometec).

#### Flow cytometric analyses

For flow cytometric analysis of surface markers, 2–3 × 10^6^ cells were used for antibody staining. Fc-block was performed in 50 µl of Fc-block antibody dilution (anti-CD16/CD32 antibody; 1:100) in FACS buffer (PBS/2% FCS/0.05% sodium azide) for 15 min on ice. Afterward, cells were washed with 800 µl of FACS buffer, centrifuged 7 min at 1400 rpm and the pellet was stained by resuspension in 50 µl antibody staining mix in FACS buffer for 20 min on ice in the dark. After the staining procedure, cells were washed with 800 µl FACS buffer, pelleted and finally resuspended in 120 µl FACS buffer for measurement (if necessary, secondary stainings were performed similarly to the primary staining and washing). For all antibody mixes, single stainings were performed for adjusting the compensation matrix. Samples were measured on a Beckman Coulter Gallios analyzer and analyzed using Kaluza software (Beckman Coulter).

#### Analyses of intestinal bacteria by flow cytometry

Intestinal content of SI, Cae and CO was scratched from the gut lumen and weight was determined on micro balance. In total, 100 mg/ml intestinal content from SI, Cae and CO was suspended in sterile PBS by intense vortexing. Fist, large particles were removed by 5 min centrifugation (2000 rpm, 4 °C). Supernatants were used for ELISA (see below). Fecal bacteria from 100 µl supernatant were washed two times by adding 1 ml sterile PBS/1% bovine serum albumin (BSA) and centrifuged at ~3200 × *g* for 5 min (in 1.5 ml Eppendorf tubes). Bacterial pallets were resolved in 25 µl blocking buffer, containing 20% normal rat serum (Stemcell, 13551) and incubated on ice. Blocking was followed by adding 50 µl of monoclonal anti-mouse IgA antibody (APC-conjugated, Clone mA-6E1, eBioscience, 17-4204-82) and 30 min incubation on ice protected from light. Bacteria were washed three times as described above. After washing, bacteria were resolved in 100 µl of 5 nM Syto9-green fluorescent nucleic acid stain (Invitrogen: S34854) in sterile PBS/1% BSA and incubated for 20 min on ice protected from light. Stained bacteria were analyzed by flow cytometry directly (no additional wash needed) and flow rate was adjusted to 12,000–15,000 cells/s by adding washing buffer. IgA staining of surface coated bacteria was measured by flow cytometry using a Gallios flow cytometer (Beckmann Coulter) with scatter adjustment for small particles. This method was adapted from ref. ^62^.

#### IgA ELISA of intestinal content

In total, 1 ml of weight-adjusted (100 mg/ml) intestinal content supernatant derived from the bacteria flow preparation (see method for analysis of intestinal bacteria by flow cytometry was centrifuged at ~3200 × *g* for 5 min to remove bacteria. Supernatant containing free SIgA was pre-diluted 1:100 in sterile PBS for anti-mouse IgA ELISA.

#### Total and sFliC-specific ELISA analysis of murine serum and feces samples

Blood was collected from cheek (living mice) or by heart puncture (sacrificed mice) and spun down in microtainers (BD Biosciences; SST Tubes; 365968) to separate serum from cellular components after 30 min of RT incubation. Feces were freshly collected from the respective mice. In total, 100 µl PBS/mg was added to feces and mixed vigorously, centrifuged 2 min with a maximum speed (13,000 rpm, Eppendorf centrifuge 5424), and the supernatant was collected and used as feces sample. Sera were diluted 1:10,000 for total Ig ELISA and 1:50 for sFliC-specific Ig ELISA in PBS 2% FCS; feces samples were diluted 1:100 in PBS 2% FCS for total Ig analysis. In total, 96-well F (flat-bottom) plates (Greiner Bio-One; 655001) were coated with either anti-IgA, anti-IgM, anti-Kappa for total Ig detection or sFliC for sFliC-specific antibody detection in ELISA coating buffer (15 mM Na_2_CO_3_ + 35 mM NaHCO_3_) at 4 °C overnight. Plates were washed 3x with PBS + 0.05% Tween-20. Plates were blocked with 200 µl PBS + 2% FCS per well at 4 °C overnight. In total, 200 µl per sample, as well as Ig standards, were applied to the ELISA plates in duplicates in row A and stepwise diluted 1:2 with PBS/2% FCS from row A to H. Final sample volume was 100 µl per well. A blank sample of PBS/2% FCS was measured on all plates. Plates were incubated for 2 h at 37 °C and then washed with PBST buffer. For detection, plates were incubated with anti-Ig (A, M or G)–Horse-radish-peroxidase (HRP)-coupled antibodies for 1 h, followed by washing and addition of 50 µl TMB substrate (OptEIA-Kit; BD Pharmingen; 555214). The color reaction was stopped with 50 µl 0.5 M sulfuric acid. The optical density of the HRP-TMB color reaction was measured on 450 nm in FLUOstar Omega ELISA-reader (BMG Labtech).

#### Immunization with soluble recombinant *Salmonella* Typhimurium flagellin

To induce mucosal IgA and systemic IgG responses in KLF2 cKO and WT cre+ control mice, we used recombinant, soluble flagellin (sFliC) for immunization^44^. Each mouse received a primary i.p. injection of 20 µg sFliC in 200 µl sterile PBS, followed by a boost immunization with 10 µg sFliC in 100 µl sterile PBS on day 35 after primary injection. Five days after boost injection, mice were sacrificed for ELISpot and flow cytometric analysis. During the time of immunization, weight of the mice was measured weekly and blood samples for ELISA analysis were collected on day 0 before immunization, on days 7, 14, 21, 28, 35 before boost immunization and on day 5 after boost.

#### Total and sFliC-specific immunoglobulin ELISpot analysis of murine antibody-secreting cells

For ELISpot analysis^63^, F-bottom Greiner 96-well plates were pre-coated as described for ELISA analysis at 4 °C overnight. After coating, plates were washed with PBS/0.05% Tween-20 and blocked with PBS/2% BSA, 200 µl per well at 4 °C overnight. Single-cell suspensions were placed on the plate in technical duplicates or triplicates and diluted 1:2 from top (row A) to bottom (row H) of the plate. For total Ig analysis, 250,000 living cells (cLP 10,000) were placed per well in row A; for sFliC-specific analysis, dilution steps started with 2 × 10^6^ living cells per well (cLP 0.5 × 10^6^) in row A. Cells were cultivated overnight on 5% CO_2_, 37 °C in the incubator in 100 µl RPMI1640/10% FCS per well. After cell cultivation, cells were disposed and plates were washed thoroughly. Washing included 1x H_2_O + 0.1% Tween-20 and 3x PBS + 0.05% Tween—repeated three times. The last washing step stayed on the plate for 10 min. For detection, plates were incubated for 1 h at 37 °C with anti-IgA-AP, anti-IgM-AP or anti-IgG-AP antibodies in 50 µl PBS/1% gelatine/1% Tween-20 per well. After secondary antibody binding, washing was repeated. AP-conjugated antibodies were detected by ESA substrate (AMP buffer, pH 10.25, containing 1 mg/ml BCIP; Sigma-Aldrich; B8503) color reaction at 4 °C overnight. Finally, plates were washed 3x with H_2_O and spots were detected on C.T.L. ELISpot reader with BioSpot Immunospot software.

#### Ig isotype-specific plasmablast and plasma cell isolation by flow cytometric cell sorting

(A) For TaqMan RNA analysis, TACI^+^/CD138^+^/IgA^+^, TACI^+^/CD138^+^/IgM^+^ and TACI^+^/CD138^+^/IgA^−^/IgM^−^ cells were stained and sorted using a MoFlo Astrios (Beckmann Coulter) cell sorter. For spleen and BM, cells of 2 C57Bl/6 mice were pooled, for mLN cells of 4 C57BL/6 mice were pooled, Fc blocked (using CD16/CD32 antibodies) in 1 ml FACS buffer and stained in 1 ml antibody staining mix, washed with 5 ml FACS buffer and resuspended for sorting in FACS buffer/0.5 mM EDTA, 30–50,000 cells per ml. Cells were sorted into 400 µl Qiazol Lysis reagent (Qiagen; 79306) for subsequent RNA isolation.

(B) For RNA-seq analysis, TACI^+^/CD138^+^/IgA^+^/B220^+^ PB/PC were sorted using a MoFlo Astrios (Beckmann Coulter) cell sorter. Three independent cell sorts were conducted using pooled cells from either mLN of two KLF2 cKO mice or mLN of two WT cre+ mice for each cell sort. For cell sorting, pooled cells from mLN of two mice were Fc blocked (using CD16/CD32 antibodies) in 1 ml FACS buffer and stained in 1 ml antibody staining mix each, washed with 5 ml FACS buffer and resuspended for sorting in FACS buffer/0.5 mM EDTA. Cells were sorted into 400 µl Qiazol Lysis reagent (Qiagen) for subsequent RNA isolation.

#### RNA isolation from sorter-purified murine plasmablasts and plasma cells

For RNA isolation, sorted cells in Qiazol Lysis reagent were resuspended in a total volume of 700 µl and homogenized with QiaShredder Mini Spin columns (Qiagen; 79654). Further RNA extraction was performed according to the Qiagen RNeasy Micro Kit protocol (Qiagen; 74004). RNA concentration was detected by NanoDrop (PeqLab) detection after blank correction.

#### cDNA synthesis from murine RNA

First-strand cDNA synthesis for TaqMan PCR was performed with Thermos Scientific RevertAid First-Strand cDNA Synthesis Kit with Oligo(dT)18 primer and 5 min, 65 °C incubation for GC rich samples (Thermo Scientific; K1622).

#### Quantitative RNA analysis by TaqMan qPCR

For quantitative RNA analysis by TaqMan PCR, 2 ng of sample cDNA + 10 µl TaqMan© Universal Master Mix II (Thermo Fisher; 427788) + 1 µl of KLF2 specific TaqMan probe (Thermo Fisher; 4331182) were diluted with 20 µl of ultra-pure H_2_O (Roth—Rotipuran; 1312.1) per detection well in a MicroAmp® Optical 96-well reaction plate (applied biosystems; N8010560). Samples were tested on KLF2 and GAPDH expression in triplicates. GAPDH was used as a housekeeping gene and for the normalization of KLF2 gene expression.

#### B cell isolation from murine spleens by magnetic cell sorting

B cell isolation from murine spleens was performed by EasySep© Mouse B cell isolation Kit (Stemcell; 19854) according to the manufacturer’s protocol. The purity of isolated B cells was verified by analyzing CD19 surface expression by flow cytometry and was routinely higher than 95%.

#### In vitro cell cultivation of murine B cells for activation and IgA class switching

Isolated B cells from murine spleens of KLF2 cKO and WT cre+ control mice were cultivated for 3 days in a cytokine mix (isotype switch mix in RPMI 1640, 10% FCS, 1 mM sodium pyruvate, 2 mM L-glutamine, 100 U/ml penicillin-streptomycin, 50 μM β-mercapto-ethanol), containing LPS (10 µg/ml), anti-CD40-antibody (10 µg/ml), IL4 (100 U/ml), IL5 (10 ng/ml), TGFβ1 (5 ng/ml) and retinoic acid (50 nM) with a starting concentration of 200,000 cells/ml and 5% CO_2_ at 37 °C (modified after^64^). Cell purity and activation status were detected via flow cytometric analysis of CD19^+^ and TACI^+^/CD138^+^ staining. Activation of B cells and class switch was analyzed on day 3 by TACI^+^/CD138^+^ and surface IgA^+^ versus surface IgM^+^ staining.

#### eFluor450 and eFluor670 labeling of in vitro generated plasmablasts

eFluor670 labeling (eBioscience; 65-0840-85): 8 × 10^6^ in vitro activated, living B cells were harvested and washed 2x with cold PBS and spun down at 1500 rpm for 5 min. Cells were stained by adding 1 ml of 2.5 µM eF670 (Thermo Fisher) in PBS to the cell pellet, resuspended and incubated for 15 min at 37 °C in a water bath. The staining was stopped by adding 10 ml of R10 media (or media with at least 10% FCS) and incubation on ice for 5 min. Then, cells were washed 3x with 10 ml of ice-cold R10 media and resuspended in 5 ml R10 medium.

eFluor450 labeling (eBiosicence; 65-0842-85): 8 × 10^6^ in vitro activated, living B cells from culture were harvested and washed 2x with cold PBS and spun down at 1500 rpm for 5 min. Cells were resuspended in 2 ml PBS. A 5 µM eF450 staining solution in PBS was prepared and 2 ml staining solution was added dropwise to the cells while agitating and incubating cells for 10 min at 37 °C in a water bath. The staining reaction was stopped by adding 20 ml of ice-cold R10 media and incubating cells for 5 min on ice. Afterward, cells were washed 2x with R10 media and finally resuspended in 5 ml R10 medium.

#### Adoptive transfer of in vitro generated class-switched plasmablasts by retroorbital i.v. injection into Rag^−/−^ recipient mice

For splenic B cell cultures, B cells were isolated using the EasySep B cell isolation kit (Stemcell) and cultured for 3 days in R10 medium supplemented with isotype switching cytokines (isotype switch mix, see above). Three days after stimulation, cell cultures of WT cre+ and KLF2-deficient B cells were analyzed for viability, activation and class-switching by flow cytometry (Fig. S4G). 8 × 10^6^ living cells per culture were harvested (cell counts and viabilities were determined by NC3000, Chemometec) and reciprocally labeled with either eFluor450 or eFluor670 proliferation dyes. Setting 1: eFluor450-labeled WT cre+ cells were equally mixed with eFluor670-labeled KLF2 KO cells. Setting 2: eFluor670-labeled WT cre+ cells were equally mixed with eFluor450-labeled KLF2 KO cells. For competitive transfer, cell mixtures of 16 × 10^6^ cells were resuspended in 75 µl sterile PBS and retroorbitally i.v injected into Rag^−/−^ recipient mice. Spleen, BM, mLN, blood and cLP of Rag^−/−^ recipient mice were analyzed by flow cytometry for the presence of transferred, labeled cells 3 days after i.v. injection to identify injected donor PB/PC within the recipient mice.

#### RNA sequencing of flow cytometric sorted mLN IgA+ plasmablasts

mLN from two animals of respective genotype (WT cre+ or KLF2 cKO) were pooled and TACI^+^/CD138^+^/B220^+^/IgA^+^ PB were FACS-sorted as described above. RNA from three pooled samples of each genotype was isolated, as described above (RIN > 7). Sequencing libraries were prepared with the Clontech SMART-Seq v4 kit (Takara) and sequenced on an Illumina HiSeq X instrument (2 × 150 bp) by Admera Health LLC. Raw reads were quality-controlled and processed with the nf-core/rnaseq pipeline^65^ with default settings. Within the pipeline, processed reads were aligned to the mouse reference genome (GRCm38.p6) with HISAT2 and summarized to gene-level counts with featureCounts^66^. Differential expression was assessed with the R package *edgeR*^67^. Genes with low expression were excluded with the *filterByExpr* function and immunoglobulin genes were removed from the analysis. Libraries were normalized with the “TMM” method before testing for differential expression between the WT cre+/KLF2 cKO samples with the *exactTest* function. Genes with a fold change > 2 and a false discovery rate ≤ 0.05 were determined as significant. Heatmaps were generated from normalized log2cpm values with the *pheatmap* package, version 1.0.12 (Raivo Kolde (2019). pheatmap: Pretty Heatmaps. R package, https://CRAN.R-project.org/package=pheatmap). Gene set enrichment analyses were performed with the *fgsea* package, version 1.12.0^68^, with pathways obtained from *reactome.db* (version 1.70.0, Willem Ligtenberg (2019) reactome.db: A set of annotation maps for reactome).

#### Histological sections and immunofluorescence staining of the small intestine and colon

The SI or CO was removed from the mouse, flushed with ice-cold PBS + 2% FCS and opened longitudinally. The gut was prepared according to the swiss-role technique: for SI: directing the ileum to the inside of the role and for the CO: directing the anus side to the inside of the role^69^. The tissue was fixated in 5 ml fresh 4% EM-grade PFA (EMS: 15710) in water for 2 h protected from light and afterwards dehydrated 2x in 10 ml 15% sucrose (J.T Baker: JTB 03483460071) solution for 8–12 h. Tissue was embedded in Cryomol containers with TissueTec® (Sakura: 4583) on dry ice and cut in the cryotome on −21 °C to 8 µm sections onto frosted slides (Thermo Scientific—Superfrost plus: J1800AMNZ). The sections of the SI and CO where rehydrated and blocked with PBS/2% FCS/2% normal rat serum/0.05% Tween-20 for 2 h. Anti-mouse IgA and J-chain staining was performed with anti-mIgA-FITC antibodies (1:200 dilution) and anti J-chain antibodies (1:100 dilution) in PBS/2% FCS/0.05% Tween-20 at 4 °C overnight. The tissue was washed 3x with up to 500 µl PBS/Tween-20. A secondary antibody staining to detect the rabbit anti-mouse J-chain IgG antibody was performed using goat anti-rabbit IgG-AF647 antibodies (1:200) in PBS/2% FCS/0.05% Tween-20 for 2 h at RT in the dark. The tissue was washed 3x with up to 500 µl PBS/Tween-20, containing DAPI (1:10000 dilution) for 10 min. Slides were covered with Fluoshield® (Sigma: F6182-20ML). All steps of the staining were performed in a wet chamber, protected from light. DAPI, FITC and AF647 fluorescence were detected using an Axioplan2 Fluorescence microscope (Zeiss) with a ×10 magnification lens. Four to five different sections along the SI and CO were pictured, showing 3–5 villi each. IgA-positive cells from the lamina propria were counted by four individuals in a blind study and average numbers of IgA-positive cells per villus were calculated. For calculation of the proportion of IgA^+^/J-chain^+^ cells in the SI and the CO, cells of 3–4 WT cre+ and 3–4 KLF2 cKO mice were counted in ×20 magnification microscope pictures.

#### Reducing and non-reducing western blot analysis for monomeric and dimeric IgA

For non-reducing western blot analyses of monomeric and dimeric IgA, serum samples were mixed 1 + 1 with 2x non-reducing loading dye (130 mM Tris-HCl (Roth, 4855.3) pH 6.8/20% glycerol (Roth, 3783.1)/4% SDS (Roth, 2326.2)/0.01% Bromophenol blue (Merck, 108122) and heated to 60 °C for 5 min. For western blot using reducing conditions, serum samples were mixed 1:5 with 5x reducing loading dye (400 mM Tris-HCl (Roth, 4855.3) pH 6.8/20% glycerol (Roth,3783.1)/10% SDS (Roth, 2326.2)/1.4 M β-mercapto-ethanol (Merck, 805740)/~0.01% Bromophenol blue (Merck, 108122) and denatured at 95 °C for 5 min. WT cre+ sera were diluted 1:2500, sera of KLF2 cKO mice were diluted 1:500 in order to get comparable amounts of IgA, due to the differences measured by ELISA (shown in Fig. S3B). Samples were loaded to a polyacrylamide gel containing 6% acrylamide (non-reducing conditions) or 10% acrylamide (for reducing conditions). Electrophoresis was carried out for 2.5 h at 250 V and 90 mA (chamber: Hoefer, power supply: SERVA). Proteins were transferred onto nitrocellulose membranes (GE Healthcare, 10600001) by semi-dry blotting (VWR Peqlab). Membranes were blocked by incubation overnight at 4 °C with 1xTBS/Tween-20 containing 5% milk (Carl Roth, T145.3). For detection of serum IgA, membranes were subsequently incubated for 1 h at room-temperature with anti-mouse IgA HRP-conjugated antibodies (Southern Biotech, 1040-05) diluted 1:10,000 in 1xTBS/Tween-20/3% BSA (Carl Roth, 8076.4). Membranes were then incubated for 1 min with ECL solution (GeneTex, GTX14698). CL-Xposure films (Thermo Scientific, 34089) were placed onto the membranes for 10 s to 30 min. After exposure, films were developed (AGFA, CP1000) and scanned. For quantification, short and long film exposures were analyzed with ImageJ (FIJI). The intensity of dimeric IgA as well as monomeric IgA bands were displayed as curves by ImageJ. The area under the curve was determined as relative value for quantification of dimeric and monomeric IgA. To calculate the ratio of relative amounts of monomeric versus dimeric IgA, Log2 ratio was calculated from area under the curve values and statistically analyzed in GraphPad Prism.

#### Transwell migration assays

For transwell assays, 5 × 10^6^ WT cre+, eF670-labeled “switch culture” blasts (day 3) were mixed with 5 × 10^6^ KLF2 cKO, eF450-labeled “switch culture” blasts (day 3) 1:1. A total number of 10 × 10^6^ cells in 500 µl medium (RPMI, containing 2% fatty acid free BSA) was added to the upper chamber of the transwell inserts (Corning®: CLS3421, 6.5 mm transwell with 5.0 μm pore polycarbonate membrane insert). To the lower chamber, either 100 nM SDF-1α (R&D: 460-SD-010/CF), 300 nM CXCL25 (R&D: 481-TK-025/CF) or 100 nM S1P (d18:1) (Avanti Polar Lipids: 860492) gradient or medium only control (MOC) were added. Plates and transwell inserts were blocked with 1% fatty acid free BSA (Sigma: A8805-5G) in PBS for 2 h on 37 °C prior to usage. Cells were washed with PBS/1% fatty acid free BSA for 30 min on 37 °C prior to the transwell migration assay. Active S1P was prepared according to the manufacturer’s protocol (Avanti) resuspended and handled in glass vessels only. Methods were adapted from^32,70^. Transwell migration was performed for 3 h at 37 °C and 5% CO_2_ incubation. Afterwards, cells were removed from the upper and lower chamber and stained with anti-CD138 and TACI antibodies for flow cytometric analysis (as described under “Flow cytometric analyses”) Basic motility was determined by calculating the ratio of CN of CD138^+^/TACI^+^ cells in the lower divided by the CN of CD138^+^/TACI^+^ cells in the upper chamber: CN lower chamber PC/CN upper chamber PC. MOC-mediated migration was set as baseline motility value. Directed migration toward a chemokine gradient must show enhanced values compared to baseline motility. WT cre+ compared to KLF2 cKO migration of CD138^+^/TACI^+^ cells was calculated by: Log2 ((CN lower/CN upper WT cre+)/(CN lower/CN upper KLF2 cKO)). MOC-mediated migration was set as baseline for active migration toward a chemokine gradient of either WT cre+ or KLF2 cKO cells.

### Quantification and statistical analysis

Statistical analysis was performed with GraphPad Prism 7.0. Depending on the size of the cohort (*n*) and comparison of one or multiple factors, within one experimental design, two-way ANOVA, *t*-test or Mann–Whitney *U* test was applied. Two-way ANOVA was used for datasets with multiple comparisons like (1) different genotypes and (2) different time points or subpopulations. A description of statistical analysis can be found in the figure legends.

**Table 1:** Key Resources Table.

Reagent or Resource	Source	Identifier
Antibodies		
Flow Cytometry		
TACI—BV421	BD Pharmingen	742840
TACI—APC	eBioscience	17-5942
TACI—PE	eBioscience	12-5942
CD138—PE.Cy7	Biolegend	142514
IgA—FITC	Southern Biotech	1040-02
IgA—AF647	Southern Biotech	1040-31
IgM—PE	Southern Biotech	1021-09
IgM—Cy5	Southern Biotech	1020-15
IgM—APC.Cy7	Biolegend	406515
IgM—Biotin	Jackson	115-065-075
B220—BV421	Biolegend	103251
B220—FITC	Biolegend	103206
B220—PE	eBioscience	12-0452-82
CD19—AF647	eBioscience	NA
CD19—APC.Fire750	Biolegend	115558
CD19—PE	eBioscience	12-0193-82
CD19—AF488	Biolegend	115521
Integrin α4 β7—Biotin	eBioscience	13-5887
CD49d—PerCP-Cy5.5	Biolegend	103619
Sav—PerCP-Cy5.5	Biolegend	405214
Sav—PerCP	BD Pharmingen	554054
Sav—APC.Cy7	Biolegend	405308
ENPP1—PE	Biolegend	149203
CCR10—APC	RD systems	FAB2815A
CCR9—PE	eBioscience	12-1991-82
CD21/35—BV421	Biolegend	123421
CD23—PE	eBioscience	12-0232-82
CXCR4 (CD184)—PE	eBioscience	12-9991
CD69—PE	Biolegend	104507
CD38—PE	Biolegend	102707
CD95—PE.Cy7	BD Pharmingen	557653
GL7—AF647	BD Pharmingen	561529
GL7—Pacific Blue	Biolegend	144614
CD16/CD32—unconjugated	eBioscience	14-0161-82
CXCR4—PE	eBioscience	12-9991
Itgβ2 (CD18)—PE	Biolegend	101407
CCR9—PE	eBioscience	12-1991-82
ItgαM (CD11b)—BV510	Biolegend	101263
Monoclonal anti-mIgA—APC (Clone mA-6E1)	eBioscience	17-4204-82
Monoclonal rabbit IgG anti-human/mouse J-chain (Clone SP105)	Invitrogen	MA5-16419
Goat anti-rabbit IgG—AF647	Jackson	111-605-144
ELISA/ELISpot/Western blot		
Kappa—unconjugated	Southern Biotech	1050-01
IgA—unconjugated	Southern Biotech	1040-01
IgA—AP	Southern Biotech	1040-04
IgA—HRP	Southern Biotech	1040-05
IgM—unconjugated	Southern Biotech	1020-01
IgM—AP	Southern Biotech	1021-04
IgM—HRP	Southern Biotech	1020-05
IgG—AP	Southern Biotech	1030-04
IgG—HRP	Southern Biotech	1030-05
IgA ĸ—unconjugated	Sigma	M-1520
IgG—whole molecule	Jackson	015-000-003
IgM ĸ—unconjugated	Sigma	M-3795
Cell culture and cell suspensions		
RPMI1640	Gibco	31870-25
Fetal Calf Sera (FCS)	Gibco	10270-106
L-Glutamine	Gibco	25030-24
Sodium-Pyruvat	Gibco	11360-039
Penicillin-Streptomycin	Gibco	15140-122
β-Mercapto-Ethanol	Gibco	31350-010
LPS	Sigma-Aldrich	L3012
αCD40 antibody (clone: FGK4.5)	BioXcell	BE0016-2
IL4	Miltenyi	130-097-761
IL5	PeproTech.	215-15-100UG
TGFβ1	R&D	7666-MB-005
Retinoic Acid (RA)	Sigma-Aldrich	R2625
SDF-1α	R&D	460-SD-010/CF
CXCL25	R&D	481-TK-025/CF
S1P (d18:1)	Avanti Polar Lipids	860492
Chemicals, Peptides, and Recombinant Proteins		
ELISA and ELISpot Coating buffer (Sodium-Carbonate-buffer)	N.A	N.A
AMP 10x Buffer (pH = 10.25) as Substrate buffer for ELISpot	N.A	N.A
BCIP (5-bromo-4-chloro-3-indolyl-phosphate, 4-toluidine salt) for ESA Substrate for ELISpot	Sigma-Aldrich	B8503
Red Blood Cell Lysis Buffer 10 x	BioLegend	420301
0.5 M EDTA (ultra-pure, pH 8.0)	Invitrogen	15575-038
1x PBS	Gibco	14190-094
BSA (Albumin Fraktion V)	Roth	8076.4
BSA (fatty acid free)	Sigma-Aldrich	A8805-5G
Percoll	GE Healthcare	17-0891-02
Soluble, recombinant *salmonella* Typhimurium Flagellin (sFliC)	provided by A.F.C., Birmingham, UK	N.A
Syto9-green fluorescent nucleic acid stain	Invitrogen	S34854
EM-grade PFA (16% stock in water)	EMS	15710
TissueTec®	Sakura	4583
Fluoshield®	Sigma	F6182-20ML
Critical Commercial Assays		
eF450 proliferation dye	eBioscience	65-0842-85
eF670 proliferation dye	eBioscience	65-0840-85
Qiazol Lysis reagent	Qiagen	79306
RNeasy Micro Kit	Qiagen	74004
RevertAid First-Strand cDNA Synthesis Kit	Thermo Scientific	K1622
KLF2 TaqMan® qPCR Assay (FAM-MGB) Mm01244979_g1	Thermo Fisher	4331182
GAPDH TaqMan® qPCR Assay (FAM-MGB) Mm99999915_g1	Thermo Fisher	4331182
TaqMan© Universal Master Mix II, no UNG	Thermo Fisher	4427788
EasySep© Mouse B cell isolation Kit	Stemcell	19854
OptEIA-Kit (TMB substrate for ELISA)	BD Pharmingen	555214
Lamina Propria Dissociation Kit, Mouse	Miltenyi	130-097-410
Solution 13, AO—DAPI (cell count and viability assay; NC3000)	Chemometec	910-3013
6.5 mm transwell with 5.0 μm pore polycarbonate membrane insert	Corning®	CLS3421
Trident femto Western HRP Substrate	GeneTex Inc	GTX14698
Deposited Data		
Raw and analyzed data	this paper	
RNA sequencing data	this paper	
Experimental Models: Organisms/Strains		
KLF2:GFP reporter mice	^25^	
KLF2 fl/wt; mb1cre+/− mice	^6^	
Rag2−/− mice	^71^	
C57BL/6 mice	Janvier	
Oligonucleotides		
KLF2 fl/wt Forward: ACTTTCGCCAGCCCGTGCGAGCG KLF2 fl/wt Reverse: TGAATTCTCGGCGCCCAGACCGTCC	Thermo Fisher	custom
Mb1-for: CTGCGGGTAGAAGGGGGTC Mb1-rev: CCTTGCGAGGTCAGGGAGCC hCre-for: ACCTCTGATGAAGTCAGGAAGAAC hCre-rev: GGAGATGTCCTTCACTCTGATTCT	Thermo Fisher	custom
Rag2 for: GACGTTCATACATGCCTTCTACCC Rag2 rev: TGTCAAATTCATCGTCACCATCAA Neo for: GGCCACACGCGTCACCTTA	Thermo Fisher	custom
Software and Algorithms		
BioSpot ® ImmunoSpot 5.1.36.	C.T.L.	https://immunospot.worldsecuresystems.com/ImmunoSpot-analyzers-software
Kaluza	Beckman Coulter	https://www.beckman.com/flow-cytometry/software/kaluza-c
Prism (7.0)	GraphPad	https://www.graphpad.com/scientific-software/prism/
NC3000 count and viability software	ChemoMetec	https://chemometec.de/zellzaehlgeraete/nc-3000-nucleocounter/
ImageJ (FIJI)	Wayne Rasband (NIH)	ImageJ (nih.gov)
Zen lite 3.4 (blue edition)	Zeiss	https://www.zeiss.de/mikroskopie/produkte/mikroskopsoftware/zen.html#downloads
Other		
7300 Real Time PCR System	Applied Biosystems	N/A
FLUOstar Omega	BMG Labtech	https://www.bmglabtech.com/de/fluostar-omega
Nanodrop ND1000 Spectrophotometer	PeqLab	http://tools.thermofisher.com/content/sfs/manuals/nd-1000-v3.8-users-manual-8%205×11.pdf
MoFlo Astrios Cell Sorter	Beckmann Coulter	https://www.beckman.de/flow-cytometry/instruments/moflo-astrios-eq
Gallios Flow Cytometer	Beckmann Coulter	https://www.beckman.de/flow-cytometry/instruments/gallios
Axioplan2 Fluorescence microscope	Zeiss	N/A
Protein Electrophoresis Unit SE600	Hoefer Inc.	
Semi-dry-blotter	VWR Peqlab	

## Supplementary information

Supplementary information

Supplementary Figures

## Data Availability

The RNA-Seq data generated during this study are available at GEO: GSE160732. Original data for figures in this paper are available on request.

## Materials availability

This study did not generate new unique reagents. There are restrictions on the availability of recombinant soluble *Salmonella* Typhimurium flagellin (sFliC) due to limited batches generated and provided by A.F.C., Birmingham, UK.
