# Supplementary material for: Krüppel-like factor 2 controls IgA plasma cell compartmentalization and IgA responses
Source: Mucosal Immunol. 2022 Mar 28;15(4):668–82. doi: 10.1038/s41385-022-00503-0 (PMC9259478; doi:10.1038/s41385-022-00503-0)
Supplement: Supplementary file 2 — Supplementary Figures [file 41385_2022_503_MOESM2_ESM.pdf]

# Supplementary Figure 1

## S1A

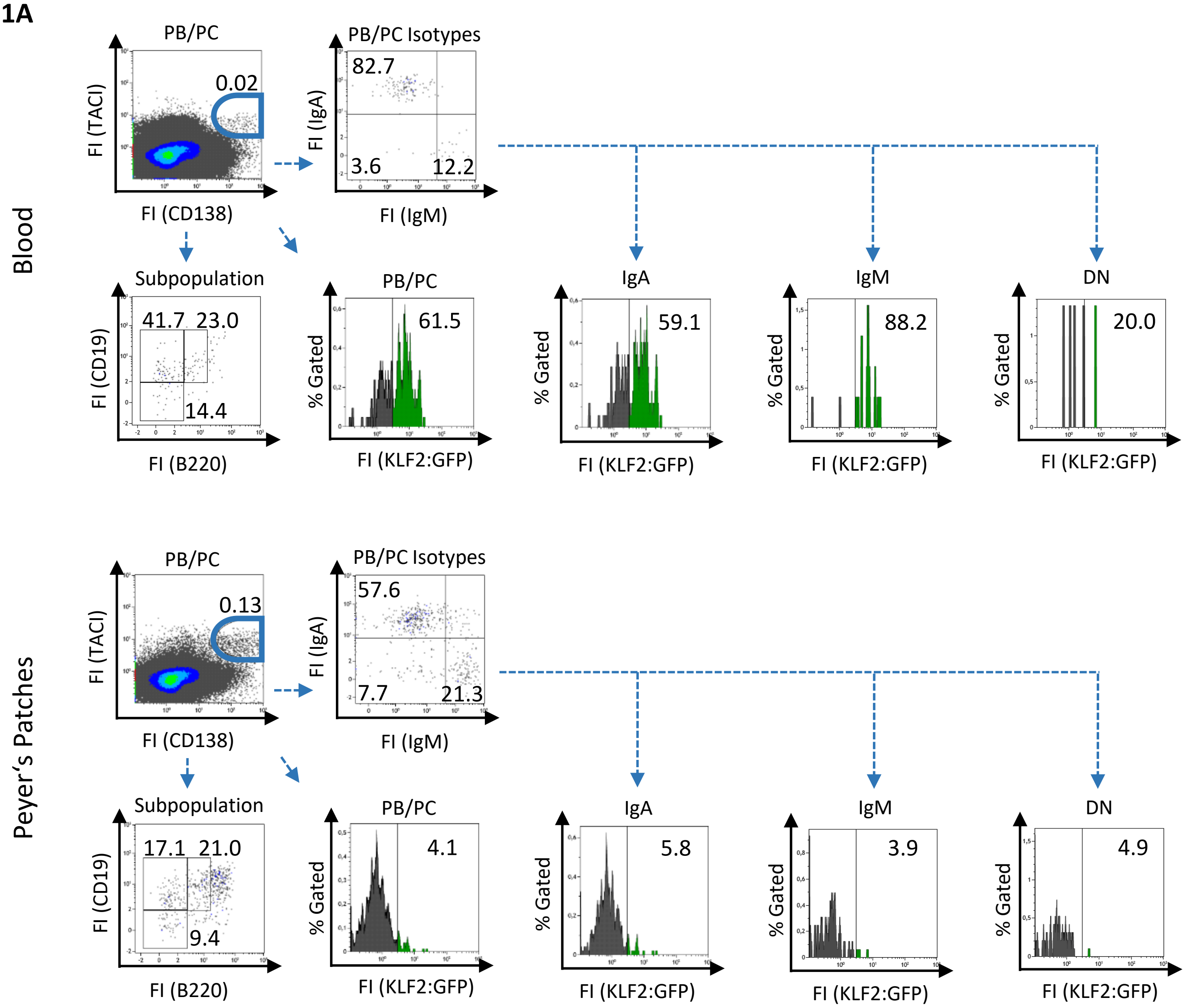


## S1B Gating strategy for PB/PC cell sorting

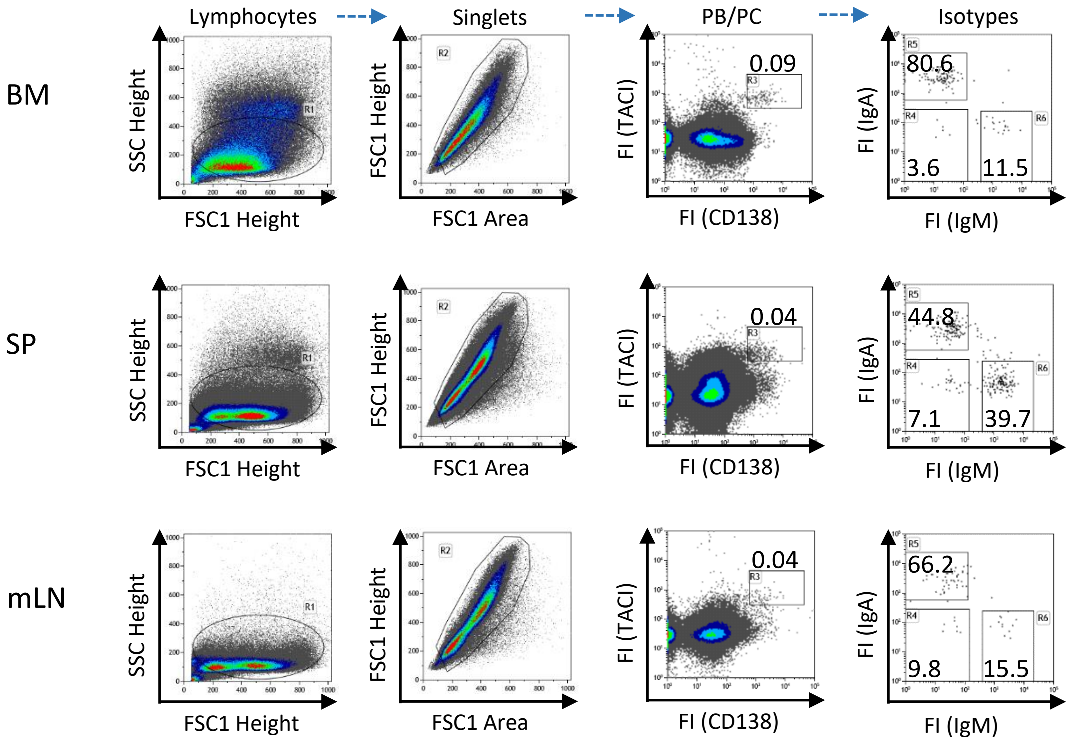

**Supplementary Figure 1:**

**(A)** Representative flow cytometric analyses of frequencies of KLF2:GFP-positive cells within gated TACI$^+$/CD138$^+$ PB/PC in blood (upper plots) and Peyer's patches (PP, lower plots), within gated TACI$^+$/CD138$^+$/IgA$^+$, TACI$^+$/CD138$^+$/IgM$^+$ and TACI$^+$/CD138$^+$/IgA$^-$/IgM$^-$ (DN) PB/PC; n=5. Numbers indicate the percentages of cells in the respective gates. For analysis of blood, flow cytometric data obtained from 5 KLF2:GFP mice was merged. (**B)** Flow cytometric gating strategy for cell sorting of CD138$^+$/TACI$^{+/}$IgA$^+$, CD138$^+$/TACI$^+$/IgM$^+$ and CD138$^+$/TACI$^+$/IgA$^-$/IgM$^-$ (DN) PB/PC of C57Bl/6 spleen (SP), bone marrow (BM) and mesenteric lymph nodes (mLN) for RNA isolation and TaqMan qPCR of KLF2 expression in SP, BM and mLN PB/PC of different IgH isotypes. FI, Fluorescence intensity; FSC, Forward Scatter; SSC, Side Scatter; GFP, green fluorescent protein.

# Supplementary Figure 2

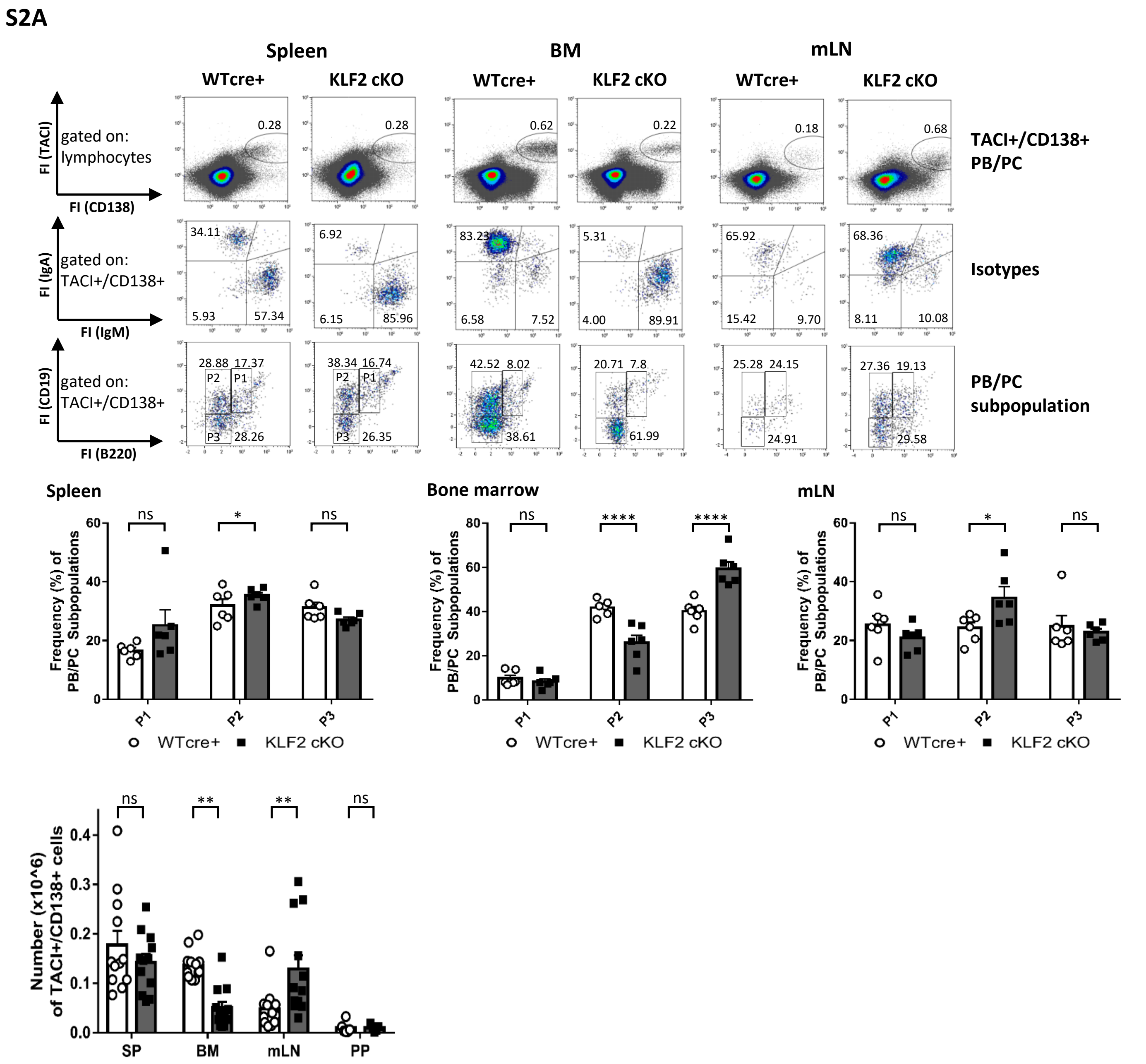


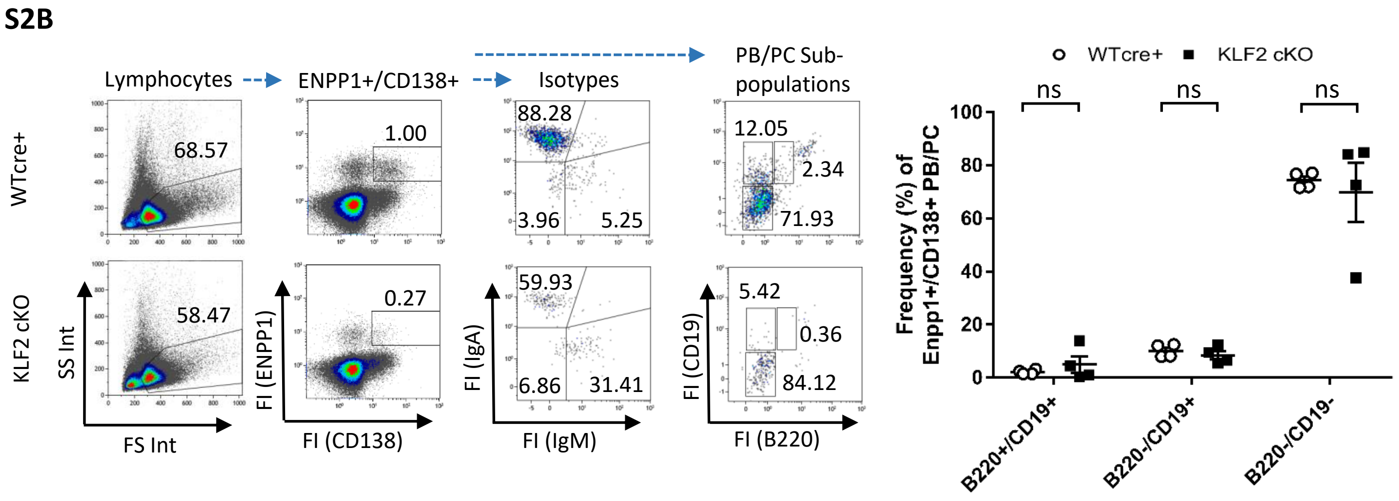

**S2C**

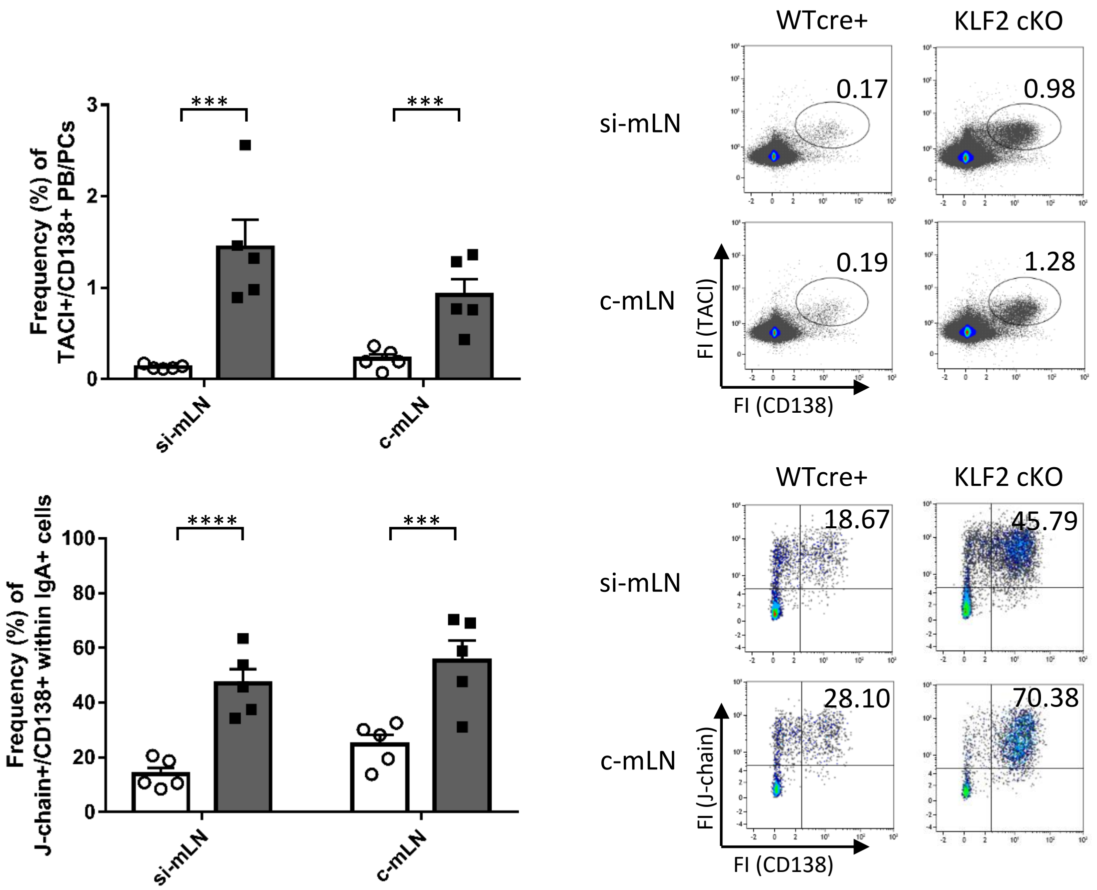


## Supplementary Figure 2:

**(A)** Flow cytometric analysis (upper part) of CD138$^+$/TACI$^+$/IgA$^+$, CD138$^+$/TACI$^+$/IgM$^+$ and CD138$^+$/TACI$^+$/IgA$^-$/IgM$^-$ (DN) PB/PC as well as in PB/PC fractions P1 (CD138$^+$/TACI$^+$/CD19$^+$/B220$^+$), P2 (CD138$^+$/TACI$^+$/CD19$^+$/B220$^{neg}$) and P3 (CD138$^+$/TACI$^+$/CD19$^{int/neg}$/B220$^{neg}$) of the SP, BM and mLN of WT cre+ controls and KLF2 cKO mice; statistical analysis (lower part) of P1, P2 and P3 PB/PC subpopulations in SP (left), BM (middle) and mLN (right) of WT cre+ control (white bars, white circles) and KLF2 cKO (grey bars, black squares) mice; numbers indicate the percentages of cells in the respective gates. n=6; mean with SEM; significance test with 2-Way-ANOVA. Additional bar charts below show the arithmetic mean with SEM of CD138$^+$/TACI$^+$ PB/PC total cell numbers in SP, BM, mLN and PP from WT cre+ mice (white circles, white bars) and KLF2 cKO mice (black squares, grey bars); N=2 experiments with n=7 (PP) and n=12 (SP, BM, mLN) mice; statistical analyses were performed for genotype comparison by unpaired t-test. **(B)** Flow cytometric analysis of CD138$^+$/ENPP1$^+$/IgA$^+$, CD138$^+$/ENPP1$^+$/IgM$^+$ and CD138$^+$/TACI$^+$/IgA$^-$/IgM$^-$ (DN) PB/PC as well as CD138$^+$/ENPP1$^+$/CD19$^+$/B220$^+$, CD138$^+$/ENPP1$^+$/CD19$^+$/B220$^{neg}$ and CD138$^+$/ ENPP1$^+$/CD19$^{int/neg}$/B220$^{neg}$ PB/PC subpopulations of the colon lamina propria (cLP) of WT cre+ controls and KLF2 cKO (left); statistical analysis of PB/PC subpopulations of the cLP (right) of WT cre+ controls (white bars, white circles) and KLF2 cKO (grey bars, black squares) mice; numbers indicate the percentages of cells in the respective gates. n=4; mean +/- SEM; significance test for PB/PC subpopulation and genotype by 2-Way-ANOVA with Sidak's correction **(C)** Flow cytometric analysis (right part) of CD138$^+$/TACI$^+$ PB/PC (upper) as well as IgA$^+$/CD138$^+$/J-chain$^+$ cells (lower) of the small intestine (si-mLN) and colon (c-mLN) draining mLN of WT cre+ controls and KLF2 cKO mice; numbers indicate the percentages of cells in the respective gates. Statistical analysis (left part) of WT cre+ control (white bars, white circles) and KLF2 cKO (grey bars, black squares) mice shows the arithmetic mean with SEM; n=5; mean with SEM; significance test with unpaired t-test. BM, bone marrow, SP, spleen; mLN, mesenteric lymph nodes; SI, small intestine; P1, PB/PC subpopulation 1; P2, PB/PC subpopulation 2; P3, PB/PC subpopulation 3; FI, Fluorescence intensity; FS, Forward Scatter; SS, Side Scatter; Int, intensity; ns, non-significant; *, $p<0.05$; **, $p<0.01$; ***, $p<0.005$; ****, $p<0.001$

# Supplementary Figure 3

**S3A**

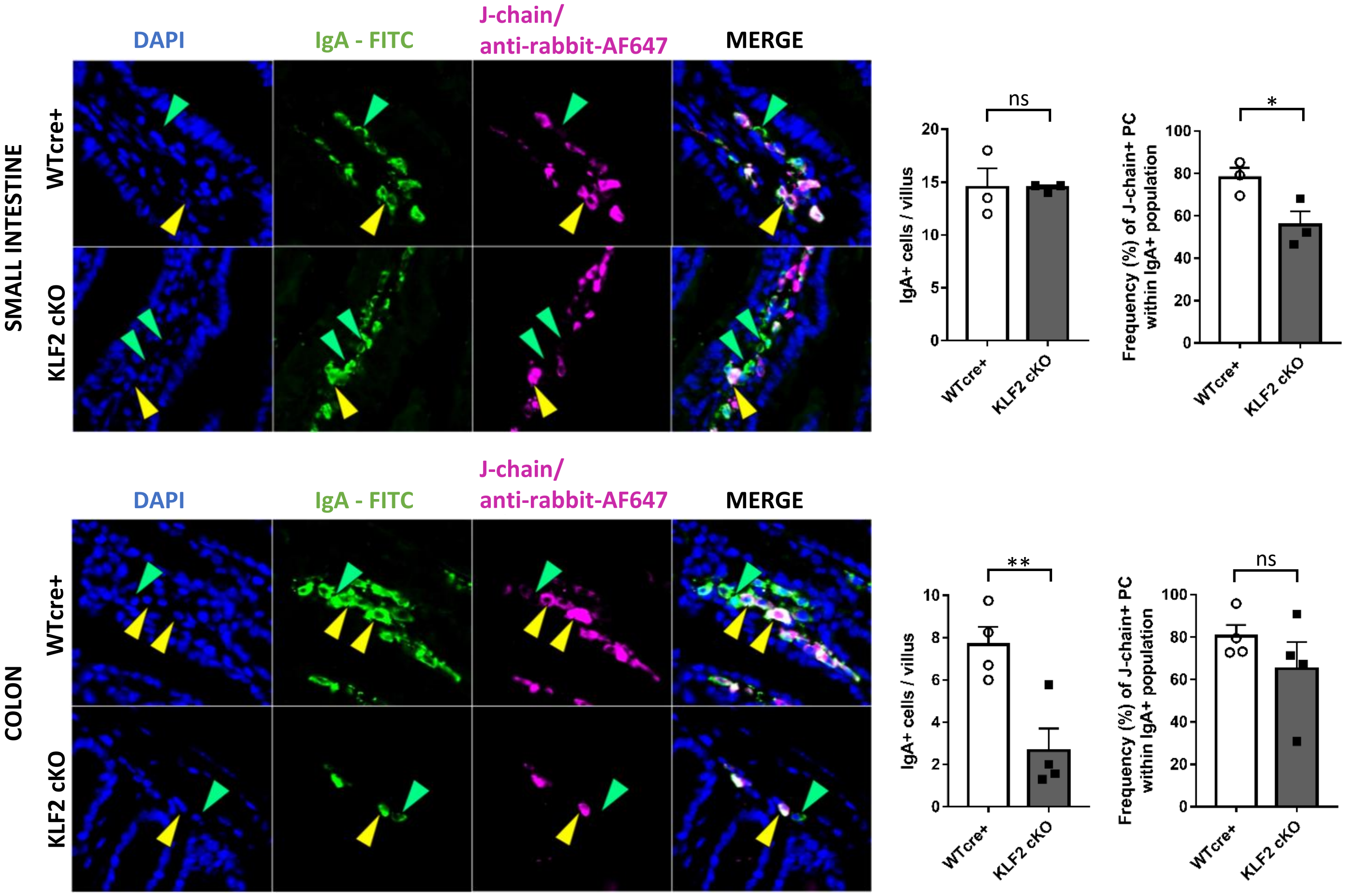


**S3B** Feces IgA ELISA

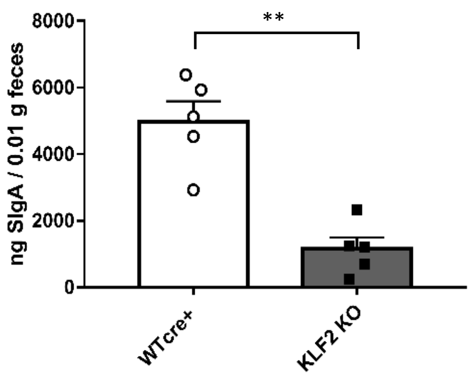

**S3C** IgA-coating of bacteria by flow cytometry

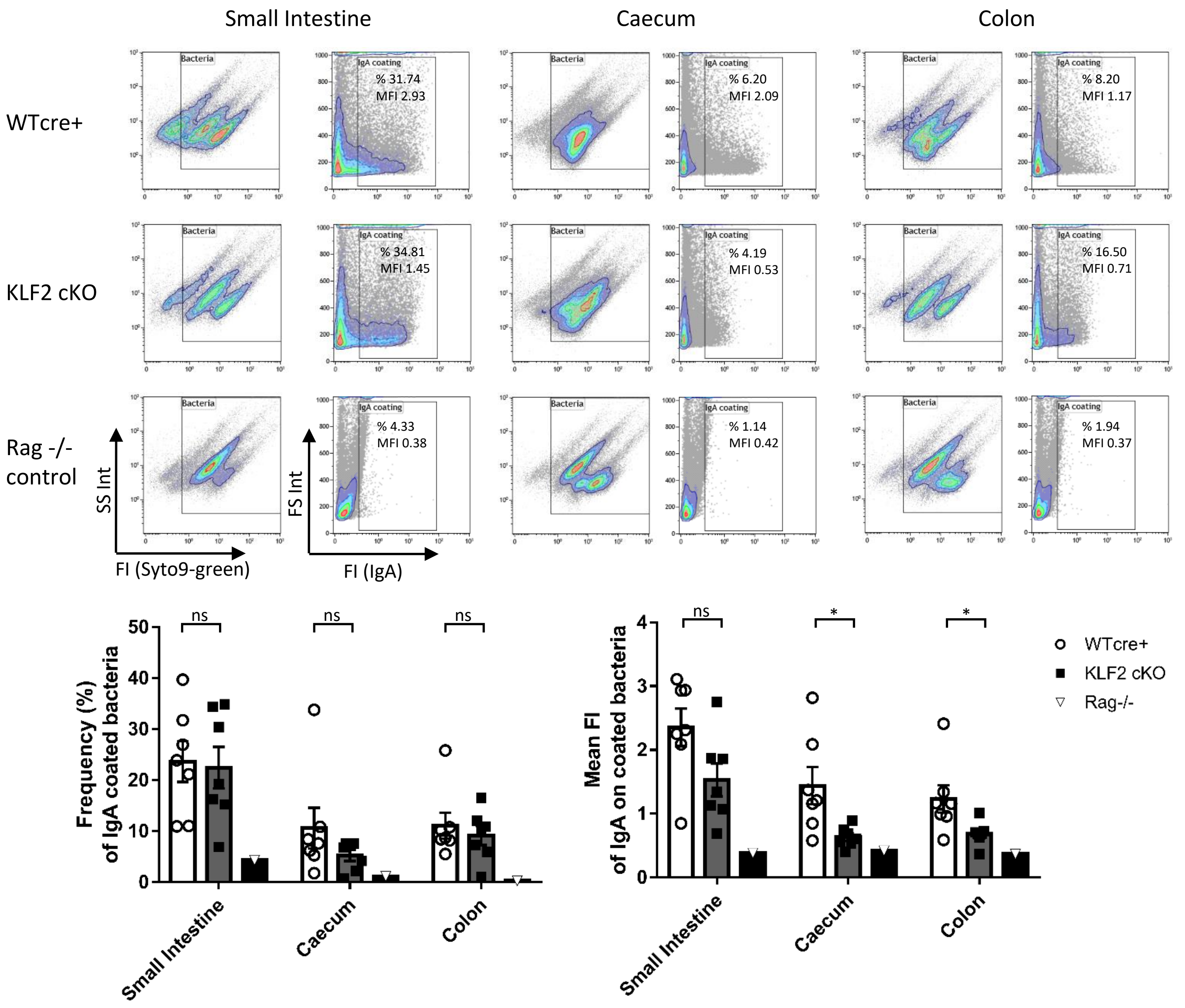


## Supplementary Figure 3:

**(A)** Immuno-histological analysis of representative sections of the small intestine (upper two rows) and colon (lower two rows) of KLF2 cKO and WT cre+ mice; IgA$^+$ cells are indicated in green, J-chain$^+$ cells are indicated in purple and DAPI positive nuclei are indicated in blue. Representative IgA$^+$ single positive cells are marked with green arrows, representative IgA$^+$/J-chain$^+$ double-positive cells are marked with yellow arrows. Statistic to right shows the arithmetic mean with SEM of IgA$^+$ (green) cells in 4-5 independent sections of the small intestine (upper left) and colon (lower left) per villus of KLF2 cKO (grey bars, black squares) and WT cre+ mice (white bars, white circles) from n=3-4 mice; Additional statistics to the right show the arithmetic mean with SEM of the proportion of IgA$^+$/J-chain$^+$ double-positive PC within the IgA$^+$ cell population of the small intestine (upper right) and colon (lower right). n=3-4 mice; statistics were performed with unpaired t-test, counting of IgA$^+$ and J-chain$^+$ cells in villi of the small intestine was independently performed by 2-4 individuals.**(B)** ELISA for secreted IgA (SIgA) in excreted feces of KLF2 cKO (grey bars, black squares) and WT cre+ control (white bars, white circles) mice, n=5; mean concentrations with SEM. Statistics were calculated with Mann-Whitney U-test **(C)** Upper panel: representative flow cytometric analysis of IgA-coating of bacteria in intestinal content (IC) of the small intestine (SI), caecum (Cae) and colon of KLF2 cKO and WT cre+ control as well as Rag$^{-/-}$ control mice. Numbers in the plots show frequencies (%) of IgA-coated bacteria and mean fluorescence intensity (MFI) of coated IgA on the bacterial surface. Lower panel: statistical analysis of frequencies (%) of IgA-coated bacteria (left) and mean fluorescence intensity (MFI) of coated IgA on the bacterial surface (right) in intestinal content (IC) of the small intestine (SI), caecum (Cae) and colon of KLF2 cKO and WT cre+ control as well as Rag$^{-/-}$ mice. N=2 experiment with n=7 mice; mean +/- SEM; statistics with unpaired 2-Way-Anova. FI, Fluorescence intensity; FS, Forward Scatter; SS, Side Scatter; Int, intensity; ns, non-significant; *, $p<0.05$; **, $p<0.01$

# Supplementary Figure 4

## S4A Sorting strategy for RNA sequencing of mLN IgA+ PB

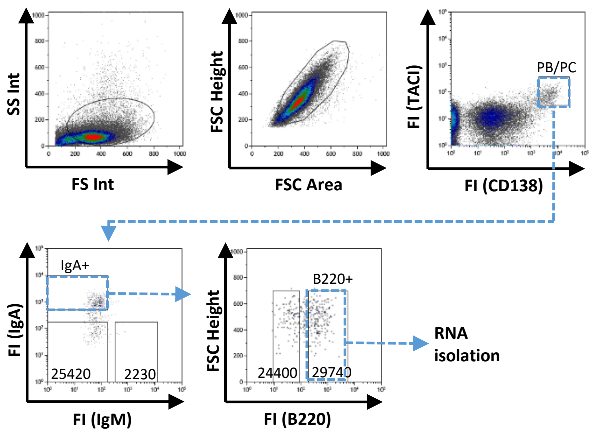


## S4B

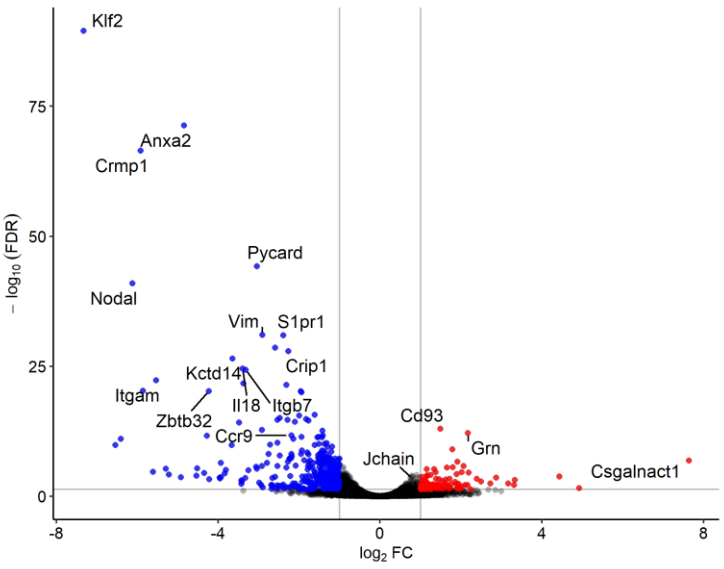


## S4C

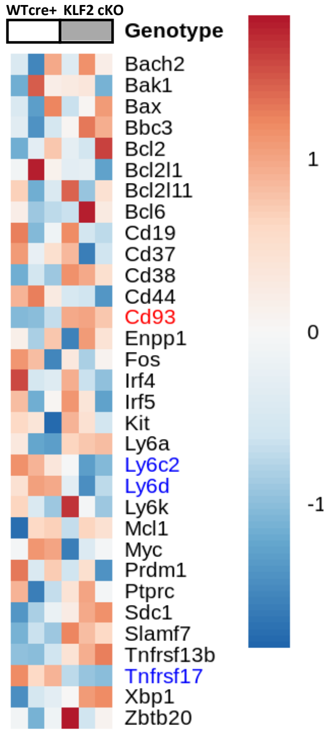


## S4D

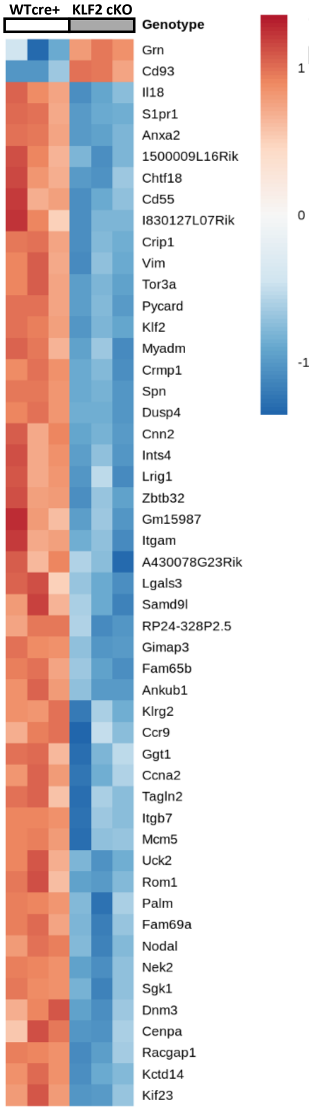


## S4E Protein abundance *in vivo*

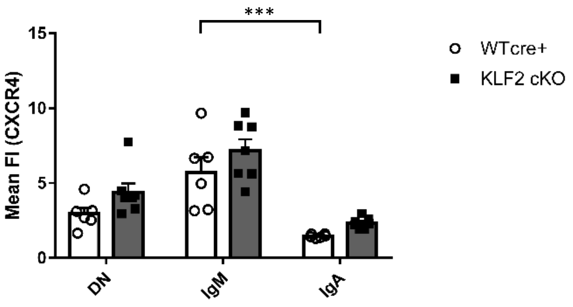

S4F

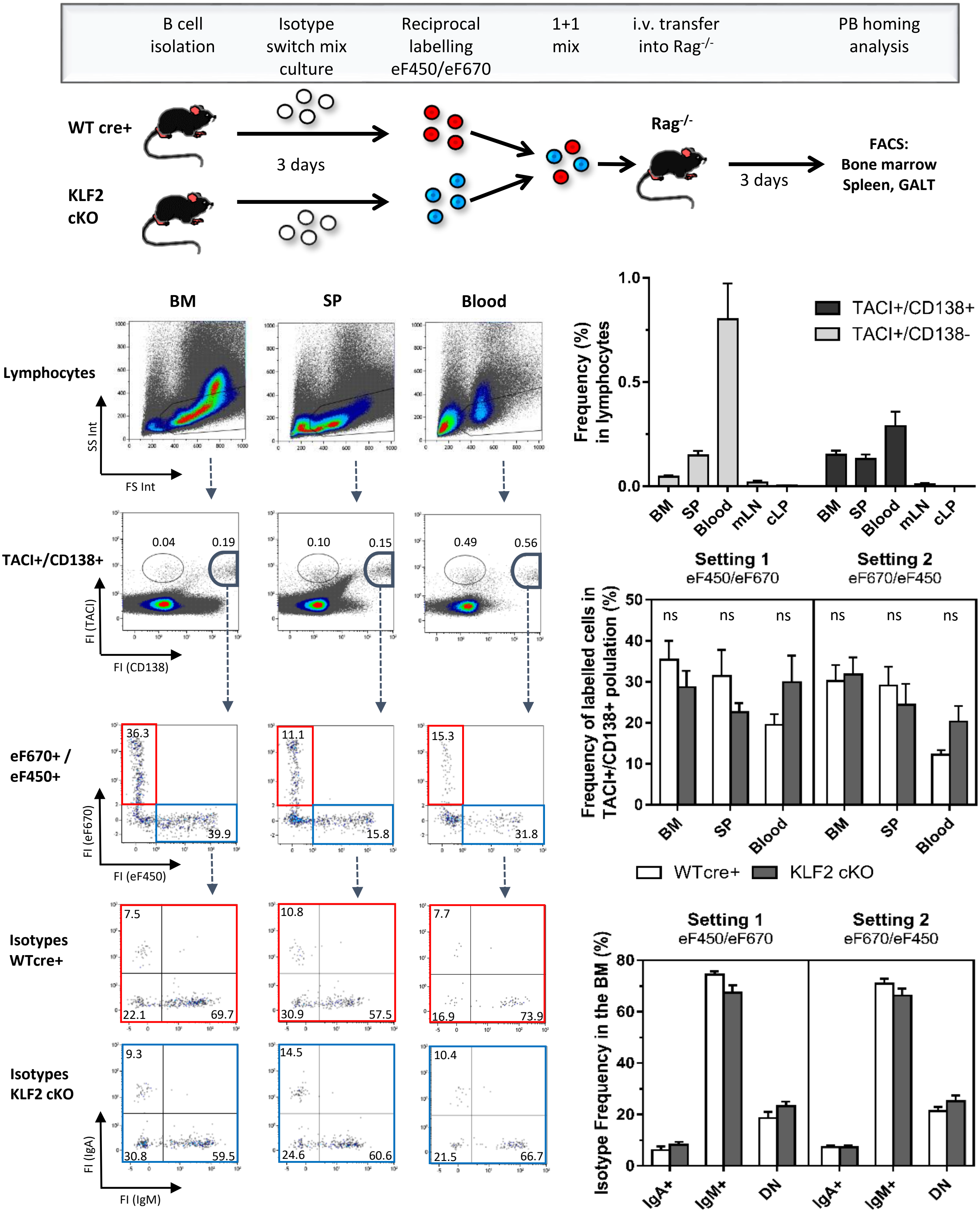

B cell isolation
Isotype switch mix culture
Reciprocal labelling eF450/eF670
1+1 mix
i.v. transfer into Rag-/-
PB homing analysis
WT cre+
KLF2 cKO
3 days
Rag-/-
3 days
FACS: Bone marrow Spleen, GALT
BM
SP
Blood
Lymphocytes
SS Int
FS Int
TACI+/CD138+
FI (TACI)
FI (CD138)
0.04
0.19
0.10
0.15
0.49
0.56
eF670+ / eF450+
FI (eF670)
FI (eF450)
36.3
39.9
11.1
15.8
15.3
31.8
Isotypes WTcre+
7.5
22.1
69.7
10.8
30.9
57.5
7.7
16.9
73.9
Isotypes KLF2 cKO
9.3
30.8
59.5
14.5
24.6
60.6
10.4
21.5
66.7
FI (IgA)
FI (IgM)
Frequency (%) in lymphocytes
TACI+/CD138+
TACI+/CD138-
BM
SP
Blood
mLN
cLP
Setting 1
eF450/eF670
Setting 2
eF670/eF450
Frequency of labelled cells in TACI+/CD138+ polulation (%)
ns
WTcre+
KLF2 cKO
Isotype Frequency in the BM (%)
IgA+
IgM+
DN

**S4G** 3 days *in vitro* activated B cells (stimulated with IgA cytokine switch mix)

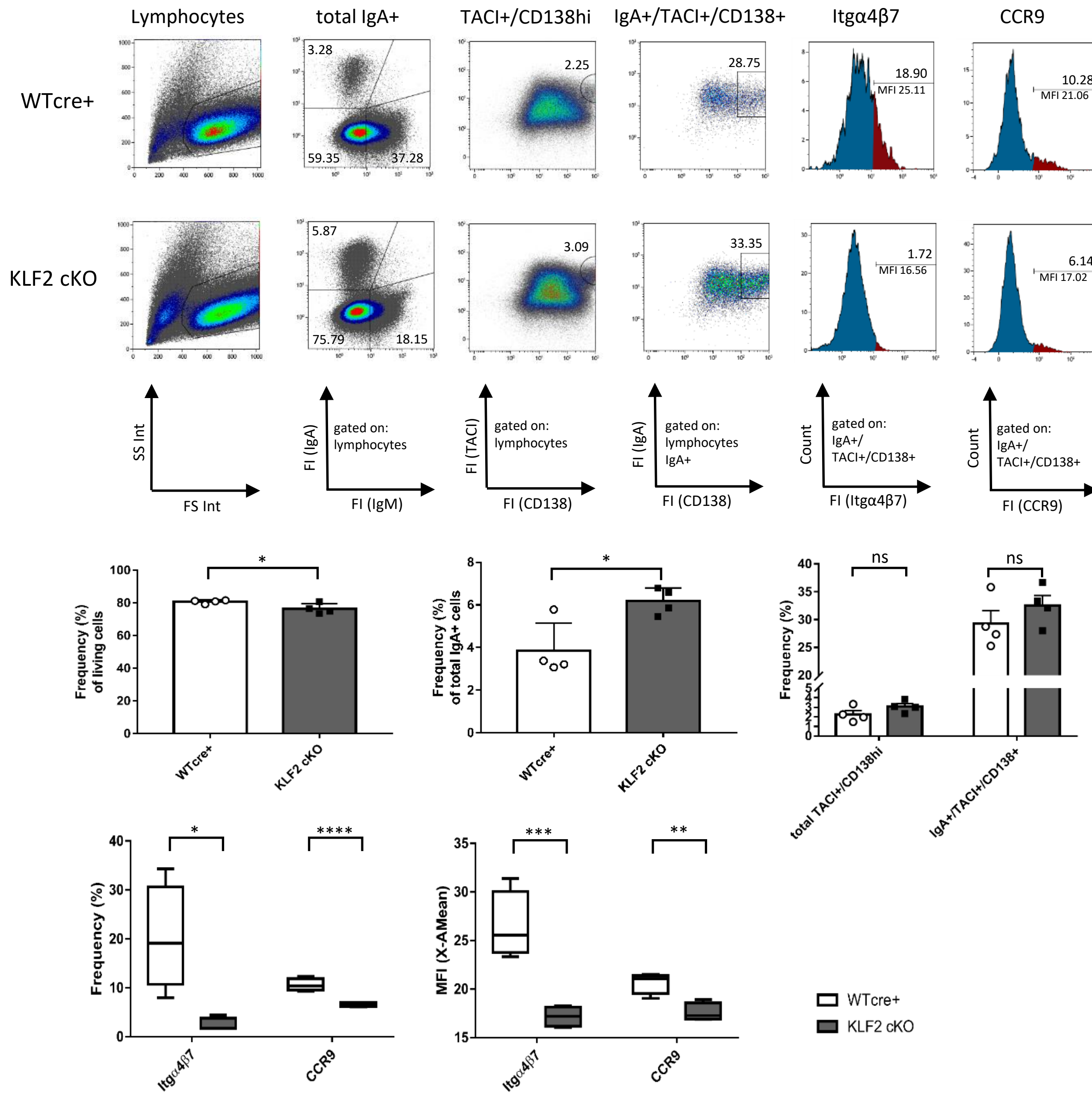


**S4H**

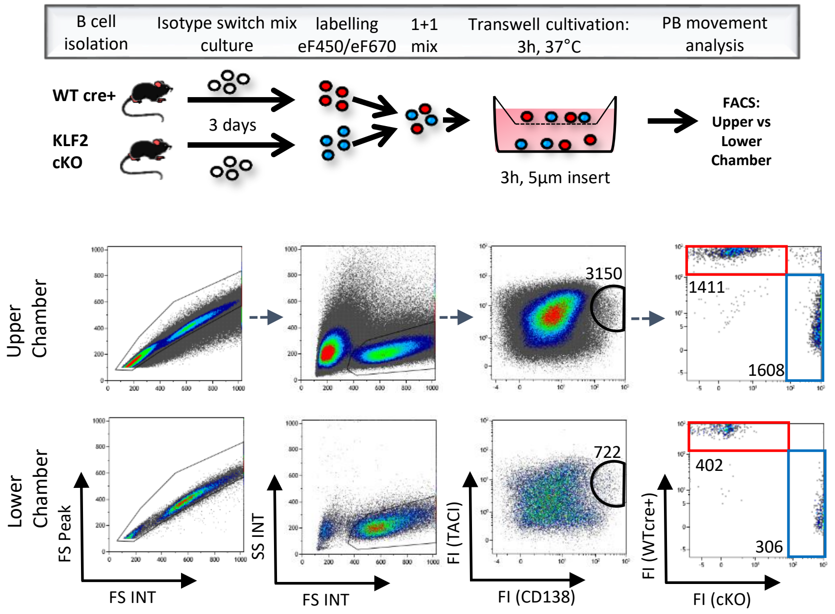

## Supplementary Figure 4:

**(A)** Flow cytometric gating strategy for TACI$^+$/CD138$^+$/IgA$^+$/B220$^+$ PB/PC sorting using an Astrios cell sorter (Beckmann Coulter) for subsequent RNA isolation and RNA sequencing by Illumina paired-end sequencing; library preparation with Clonetech: SMARTseq V4; numbers indicate representative cell numbers of sorted, collected populations. **(B)** Volcano Plot with upregulated (log2FC > 1, FDR ≤ 0.05) and downregulated genes (log2FC < -1, FDR ≤ 0.05) in KLF2 cKO compared to WT cre+ samples and **(C)** Heatmap of plasma cell signature gene expression identified by RNA-seq analysis in TACI$^+$/CD138$^+$/IgA$^+$/B220$^+$ sorter-purified PB from the mLN of KLF2 cKO (KO) and WT cre+ control (WT) mice. n=3 (6 mice per genotype, sorted cells from two mice were pooled). **(D)** Heatmap of the top 50 significantly regulated genes identified by RNA-seq analysis in TACI$^+$/CD138$^+$/IgA$^+$/B220$^+$ sorter-purified PB from the mLN of KLF2 cKO and WT cre+ control mice. (**E)** Statistics of flow cytometric analysis of CXCR4 mean fluorescence intensities on surface of WT cre+ control (white bars, white circles) and KLF2 cKO (grey bars, black squares) TACI$^+$/CD138$^+$/IgA$^+$, TACI$^+$/CD138$^+$/IgM$^+$, TACI$^+$/CD138$^+$/IgA$^-$/IgM$^-$ (DN) PB/PC of the mLN; n=6-7; mean with SEM; statistics for isotype and genotype analysis by 2-Way-Anova with Sidak's correction. **(F) Upper part**: schematic overview of the workflow; in vitro activation and class switch of splenic B cells was analyzed on day 3 by flow cytometry; 8x10$^6$ cultured KLF2 cKO and WT cre+ cells were reciprocally labeled with eFluor670 or eFluor450 proliferation dyes and mixed in equal parts before injection (Setting 1: eFluor450-labeled WT cre+ cells were mixed with eFluor670-labeled KLF2 cKO cells; Setting 2: eFluor670-labeled WT cre+ cells were mixed with eFluor450-labeled KLF2 cKO cells); 16x10$^6$ mixed cells were injected i.v. into Rag$^{-/-}$ recipient mice. BM, SP, blood, mLN and cLP of recipient mice were analyzed 3 days after injection by flow cytometry; **Lower part (left):** Flow cytometric analysis of transferred, eFluor-labeled CD138$^+$/TACI$^+$ PB/PC in the BM (upper), SP (middle) and Blood (lower) cells of Rag$^{-/-}$ recipient mice. FACS plots from left to right: lymphocyte gating, gating for CD138$^+$/TACI$^+$ PB/PC, gating for eFluor450-labeled KLF2 cKO (blue rectangles) and eFluor670-labeled WT cre+ (red rectangles) cells, gating for isotype distribution within the respective labeled cell subsets. Numbers indicate the percentage of cells in the respective gates. **Lower right:** The upper right statistic shows the arithmetic mean with SEM of the distribution (%) of TACI$^+$/CD138$^-$ (activated cells, light grey bars) and TACI$^+$/CD138$^+$ PB/PC (dark grey bars) in BM, SP, Blood, mLN and cLP of Rag$^{-/-}$ recipient mice on day 3 after injection. Below, arithmetic mean with SEM of injection-matched frequencies of either eFluor450 or eFluor670-labeled, TACI$^+$/CD138$^+$ PB/PC and TACI$^+$/CD138$^+$/IgA$^+$, TACI$^+$/CD138$^+$/IgM$^+$ and TACI$^+$/CD138$^+$/IgA$^-$/IgM$^-$ (DN) isotype distribution within the labeled PB/PC subsets is depicted next to the representative gates. Comparison of WT cre+ (white bars) and KLF2 cKO (grey bars) cell frequencies in BM, SP and Blood (setting 1: left; setting 2: right); n=4 per setting, statistics for organ (upper) or isotype (lower) and genotype comparison by 2-Way-ANOVA with Sidak's correction for multiple comparisons. **(G)** Flow cytometric analysis of class switch mix-activated WT cre+ and KLF2 cKO B cell cultures after 3 days of cultivation; gated plots (upper) and statistical data (lower) for viability (left) by FS Int vs. SS Int. frequencies of IgA$^+$ living cells (middle), activation status defined as TACI$^+$/CD138$^{hi}$ (right) for total as well as IgA$^+$ cells. Itgα4β7 and CCR9 surface expression on IgA$^+$/TACI$^+$/CD138$^+$ PB/PC (below), cell frequencies (left) and surface mean fluorescence intensities (MFI) (right); mean with SEM; n=4; statistical analysis with unpaired t-test. **(H)** Schematic overview of the workflow and gating strategy of competitive transwell migration assays; *in vitro* activation of splenic B cells was assessed on day 3 by flow cytometry; equal living numbers of cultured KLF2 cKO and WT cre+ cells were labeled with eFluor670 (WT cre+) and eFluor450 (KLF2 cKO) proliferation dyes and mixed before 3 h cultivation in chambers with 5 µm transwell insert. Upper and lower chamber were analyzed for labeled cells and their activation status by flow cytometry. FI, Fluorescence intensity; FS, Forward Scatter; SS, Side Scatter; Int, intensity; ns, non-significant; *, p<0.05; **, p<0.01; ***, p<0.005; ****, p<0.001
